# Role of Matricellular Proteins in Endothelial Cell Inflammation and Atherosclerosis

**DOI:** 10.3390/antiox14111338

**Published:** 2025-11-06

**Authors:** Ravi Varma Aithabathula, Santosh Kumar, Bhupesh Singla

**Affiliations:** Department of Pharmaceutical Sciences, College of Pharmacy, The University of Tennessee Health Science Center, Memphis, TN 38103, USA; raithaba@uthsc.edu (R.V.A.); ksantosh@uthsc.edu (S.K.)

**Keywords:** atherosclerosis, matricellular proteins, endothelial cells, inflammation, reactive oxygen species, autophagy

## Abstract

The vascular endothelium serves as a critical barrier preventing the transmigration of monocytes, circulating lipoproteins, and other molecules into the subendothelial space, and plays a vital role in regulating vascular tone. A dysfunctional and inflamed endothelial layer in response to disturbed blood flow or other proatherogenic risk factors is the initiating event in the pathogenesis of atherosclerosis, suggesting the importance of an intact and properly functioning endothelium in preventing the onset and progression of this disease. Accumulated evidence demonstrates the significant role of matricellular proteins, which are non-structural and secretory extracellular matrix (ECM) proteins, in the development of atherosclerosis. These proteins exert multifaceted effects on endothelial cells (ECs) ranging from reactive oxygen species (ROS) production, endoplasmic reticulum stress, and expression of adhesion molecules to autophagy and compromised barrier function via stimulating various molecular mechanisms. Given the critical roles of these processes in EC function and atherosclerosis, a better understanding of signaling pathways governed by matricellular proteins in ECs is required to develop therapeutic strategies for suppressing or preventing atherosclerosis and related cardiovascular diseases (CVDs). This review comprehensively summarizes the existing literature on the diverse roles of matricellular proteins in regulating EC inflammation and function, and highlights their potential as viable therapeutic targets for maintaining vascular health and inhibiting the progression of atherosclerosis.

## 1. Introduction

Atherosclerosis is a chronic inflammatory vascular disease characterized by the buildup of cholesterol-rich plaques that progressively narrow arterial blood vessels and reduce blood flow to vital organs like the heart and brain. This pathology is a key underlying cause of various CVDs, including myocardial infarction, ischemic cardiomyopathy, stroke, and peripheral vascular disease, primarily due to arterial occlusion by plaques or embolism from ruptured plaques [1]. In 2022, the age-adjusted mortality rate attributable to CVDs in the United States was 224.3 per 100,000 [2]. 

A better understanding of the pathogenesis of atherosclerosis is important for the development of effective therapies to prevent and treat this complex vascular disease. Each stage of atherogenesis is marked by distinct histological features and cellular processes. Early stages, beginning in childhood, involve the accumulation of low-density lipoprotein (LDL) cholesterol in the arterial intima, leading to endothelial inflammation and the recruitment of immune cells [3]. As the disease progresses, monocyte-derived macrophages and vascular smooth muscle cells (VSMCs) contribute to plaque formation through lipid uptake, foam cell formation, ECM accumulation, and cell death. The stability of the plaque’s fibrous cap decreases over time, increasing the risk of rupture and subsequent thrombosis, which can result in severe cardiovascular events such as myocardial infarction [4,5,6]. Understanding the underlying molecular mechanisms is essential for developing targeted strategies to intervene at various stages of atherosclerosis, thereby improving outcomes for patients at risk of cardiovascular complications. ECs play a crucial role in the development of atherosclerosis by responding to various atherogenic stimuli present in the bloodstream, including increased lipid and glucose levels, elevated blood pressure, and inflammatory molecules/cells. These stimuli influence EC phenotypes, which are implicated in various stages of atherosclerotic lesion development. Several genetic studies have demonstrated the involvement of EC dysfunction in the pathogenesis of atherosclerosis [7,8,9,10]. However, our knowledge of the exact mechanisms responsible for EC dysfunction regulating atherogenesis is far from being completely understood and requires future investigations. Earlier studies have suggested the important roles of matricellular proteins in the development of atherosclerosis [11,12]. This review summarizes various types of matricellular proteins and their contributions to endothelial dysfunction and the development of atherosclerosis.

## 2. Role of Vascular Endothelium in Atherogenesis

Atherosclerosis, a chronic inflammatory disease affecting large- and medium-sized arteries, involves intricate interactions among different cell types, including vascular ECs, VSMCs, macrophages, T cells, and B cells. Additionally, it is accompanied by increased levels of lipoproteins, ECM constituents, and various pro-inflammatory cytokines and chemokines [13]. Both genetic and environmental factors regulate the initiation and progression of atherosclerosis. These factors include dietary patterns, adiposity, tobacco use, diabetes, hypertension, and lipidemia, which collectively regulate the expression and function of different proteins involved in lipid metabolism, inflammatory processes, oxidative stress, and immune responses. One significant determinant increasing the risk of atherosclerosis is the levels of small, dense LDL particles. These particles possess heightened atherogenic properties compared to larger, more buoyant LDL counterparts, due to increased arterial infiltration and entrapment, elevated susceptibility to oxidative and glycation modifications, aggregation tendency, reduced affinity for LDL receptors, and higher capacity to induce pro-inflammatory and prothrombotic events [14].

Vascular ECs are situated at the critical interface between semi-solid tissues and circulating blood, and play pivotal roles both in physiological and pathophysiological processes, particularly in the development of atherosclerosis. These roles include—(a) regulating the entry and retention of lipoproteins and leukocytes into the subendothelial space of the arterial wall, which are the main components of plaque [15], (b) producing and secreting various factors such as nitric oxide, cytokines, chemokines, and adhesion molecules, which regulate vascular tone, inflammation, oxidative stress, and coagulation [16,17,18,19,20,21], (c) changing their proliferation rate, turnover, metabolism, permeability, and plasticity, which can alter the structure and function of the vessel wall [22], (d) interacting with other cell types, such as VSMCs, macrophages, monocytes, T cells, and B cells, to modulate the immune response and the stability of the plaque [13,23], (e) contributing to the resolution of inflammation, thereby preventing or limiting the complications related to plaque formation. 

Vascular EC inflammation refers to a condition where the endothelium layer of blood vessels becomes damaged or dysfunctional, potentially leading to several vascular diseases such as atherosclerosis, hypertension, and coronary artery disease (CAD). The risk factors contributing to endothelial dysfunction include diabetes, high blood pressure, smoking, obesity, and aging. These factors can reduce the production or availability of nitric oxide (NO), a crucial molecule for maintaining vascular homeostasis, and elevate the levels of various pro-inflammatory cytokines, and expression of various immune cell adhesion molecules or lipoprotein receptors on the surface of ECs [24,25,26,27]. Here, we provide a brief overview of various types of EC inflammation and the signaling pathways. These aspects have been comprehensively reviewed in previous publications [7,10,26].

### 2.1. Endothelial Activation

**Figure 1:**
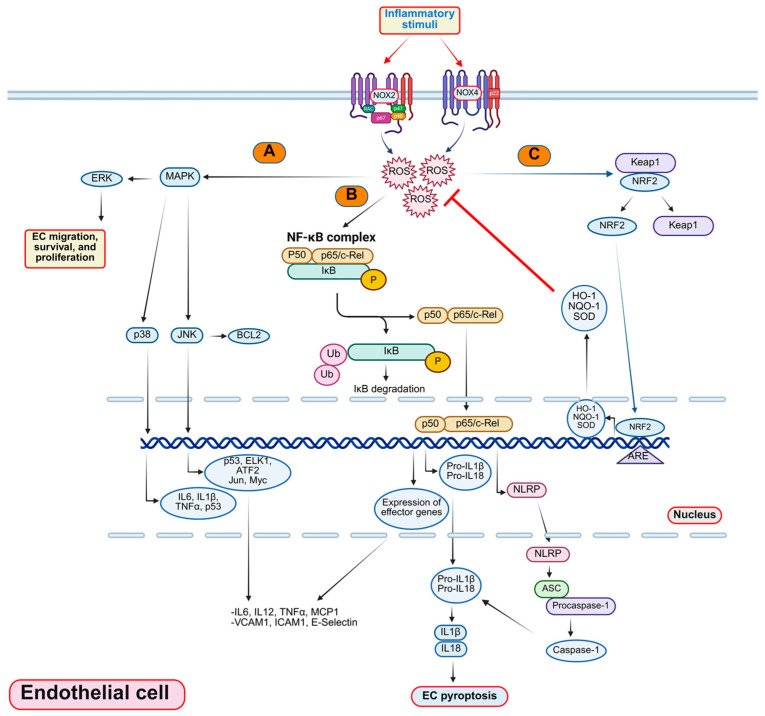
Key signaling pathways governed by ROS and/or matricellular proteins in EC inflammation: (**A**) MAPK/p38 pathway: The activation of the Mitogen-Activated Protein Kinase (MAPK) pathway, comprising ERK, JNK, and p38 subfamilies, stimulates the expression of various molecules, including IL6, IL1β, TNFα, p53, ELK1, ATF2, MYC, VCAM1, ICAM1, and E-selectin, in ECs [51,52]. ERK promotes cell survival, proliferation, and migration JNK induces cell death and inflammation, and p38 contributes to inflammation and apoptosis [53]. Crosstalk between these subfamilies regulates cellular responses, affecting processes critical to atherosclerosis, such as proliferation, differentiation, migration, and apoptosis. (**B**) NF-κB pathway and NLRP3 inflammasome: The NF-κB pathway regulates the expression of inflammatory genes in ECs. ROS-induced activation of IκB kinase (IKK) leads to the degradation of the inhibitory protein IκB, allowing nuclear translocation of NF-κB (p50/p65 heterodimer). Once in the nucleus, p50/p65 heterodimer activates the transcription of pro-inflammatory genes IL-6, IL-12, and TNFα, and adhesion molecules (VCAM1, ICAM1, and E-selectin) [54,55]. NLRP3 inflammasome, which senses ROS and danger signals, contributes to inflammatory responses in ECs. ROS or other stimuli activate NLRP3, forming a complex with the adaptor protein ASC and pro-caspase-1. Caspase-1 activation cleaves pro-IL1β and pro-IL18 into their active forms, inducing EC pyroptosis (a form of inflammatory cell death). This process amplifies inflammation within atherosclerotic arteries, potentially influencing plaque stability and progression [56,57,58]. (**C**) Nrf2 pathway: Counteracting the pro-inflammatory milieu, Nuclear Factor Erythroid 2-Related Factor 2 (Nrf2) pathway functions as a major regulator of antioxidant gene expression. ROS-triggered modification of Kelch-like ECH-associated protein 1 (Keap1) leads to the release and stabilization of Nrf2 [59]. Nuclear translocation of Nrf2 activates antioxidant response element (ARE)-mediated transcription of antioxidant genes such as heme oxygenase-1 (HO-1), NAD(P)H quinone oxidoreductase 1 (NQO1), and superoxide dismutase (SOD) [60]. This antioxidative arm plays a crucial role in protecting ECs against oxidative stress, a hallmark of atherosclerosis [61]. Beyond these pathways, molecules such as Vascular Endothelial Growth Factors (VEGF) [62], Transforming Growth Factor-beta (TGFβ) [63], IL6 [37], and Platelet-Derived Growth Factor (PDGF) activate various signaling cascades and regulate angiogenesis, vascular remodeling, and inflammation, further shaping the atherosclerotic landscape [64].

This type of EC inflammation occurs in response to various pro-inflammatory cytokines, such as tumor necrosis factor-alpha (TNF-α), interleukin-1 (IL-1), and IL-6, or endotoxins like lipopolysaccharide (LPS). Endothelial activation leads to an upregulation in the expression of adhesion molecules, including E-selectin, intercellular adhesion molecule-1 (ICAM-1), and vascular cell adhesion molecule-1 (VCAM-1), and increased secretion of pro-inflammatory chemokines/cytokines, which compromise endothelial barrier function, resulting in increased vascular permeability (Figure 1). This facilitates the recruitment, adhesion, and transmigration of leukocytes across the endothelial layer [28]. Furthermore, endothelial activation induces the expression of pro-coagulant factors, such as tissue factor and von Willebrand factor, while concurrently downregulating anti-coagulant factors, such as thrombomodulin and prostacyclin. This imbalance increases the risk of thrombus formation [29]. Endothelial activation plays an important role in the pathogenesis of various inflammatory diseases, such as atherosclerosis, sepsis, and rheumatoid arthritis [30,31]. The major signaling pathways involved in endothelial activation include nuclear factor-kappa B (NF-κB), MAPK, and PI3K/Akt pathways [32,33,34] (Figure 1 and Table 1).

**Table 1:** Major markers of EC inflammation.

Name of the Marker	Function	Refs.
C-reactive protein (CRP)	CRP is produced by the liver in response to inflammation. CRP binds to damaged ECs and exacerbates inflammation and oxidative stress. In addition, it reduces NO production, increases endothelin-1 synthesis, and suppresses endothelium-dependent arterial relaxation.	[35,36]
IL-6	IL-6 is a pro-inflammatory cytokine secreted by ECs and other cells during inflammation. It plays an important role in upregulating the production of CRP and other inflammatory mediators. It also promotes the expression of chemokines and adhesion molecules on ECs, which aids in leukocyte recruitment.	[35,37]
VCAM-1	VCAM-1, an adhesion molecule, binds to integrins found on leukocytes, facilitating their attachment and transmigration across the endothelium. VCAM-1 expression on ECs increases in response to various inflammatory stimuli.	[38,39]
E-selectin	It interacts with sialylated glycoproteins on leukocytes, enabling leukocyte rolling and initial attachment to the endothelium.	[35,40]
ICAM-1	Similar to VCAM-1, it helps in leukocyte attachment and transmigration across the endothelium.	[35,41]
Endothelin-1	It is a potent vasoconstrictor and pro-inflammatory agent synthesized by ECs. It can stimulate the expression of adhesion molecules and chemokines on ECs. Additionally, it activates the NF-κB pathway, a key regulator of inflammation.	[35,42]
MCP-1/CCL2	ECs and VSMCs secrete MCP-1, which plays a crucial role in attracting monocytes and macrophages to the subendothelial cell layer. This recruitment process facilitates the accumulation of lipids within these immune cells, ultimately contributing to the formation of atherosclerotic lesions.	[35,43,44]
TNF-α	TNF-α, originally identified for its anti-tumor properties, is a key pro-inflammatory cytokine associated with various CVDs. TNF-α contributes to endothelial dysfunction via promoting oxidative stress and reducing NO production, thus impairing endothelium-dependent vasodilation across different vascular beds.	[35,45]
Interleukin-1 β (IL-1β)	IL-1β promotes the development of early atherosclerotic lesions by enhancing the adherence of monocytes to ECs through the elevation of adhesion molecules.	[35,46]
Transforming growth factor β (TGF-β)	TGF-β induces the expression of several pro-inflammatory chemokines, cytokines, their receptors, and adhesion molecules on ECs. Moreover, it stimulates the expression of matrix metalloproteinases and fibronectin (FN), which are closely associated with inflammation.	[47]
Interleukin-18 (IL-18)	IL-18 belongs to the IL-1 cytokine family and was originally found in macrophages and Kupffer cells. IL-18 triggers IFN-γ production by T cells.	[35]
CD40/CD40L	CD40L, a TNF family member, and its receptor CD40 are co-expressed in activated T lymphocytes, ECs, SMCs, and macrophages in atherosclerotic lesions.	[35]
Interleukin-8 (IL-8)	IL-8 is a pro-inflammatory cytokine that plays a significant role in EC inflammation, particularly in the context of CVDs. IL-8 functions through its receptors, CXCR1 and CXCR2, present on circulating immune cells, particularly neutrophils, leading to their chemotaxis and adhesion to the EC surface. IL-8 also plays a role in promoting ICAM-1 and VCAM-1 expression on ECs.	[48,49]
CXCL12	CXCL12, also known as stromal cell-derived factor 1 (SDF-1), is a chemokine that plays a crucial role in vascular development, tissue repair, and inflammation. In ECs, CXCL12 is involved in various processes: EC migration, inflammation, and angiogenesis.	[50]

### 2.2. Endothelial Dysfunction

Endothelial dysfunction arises due to impairment in the vasoprotective function of the endothelium, including vasodilation, anti-inflammation, and anti-oxidation [65]. It is primarily caused by the reduced production and/or bioavailability of NO [7], a key EC-derived relaxing factor that modulates vascular tone, inhibits platelet aggregation, and suppresses leukocyte adhesion. Endothelial dysfunction is closely linked to the development and progression of CVDs—hypertension, CAD, and stroke. It represents the earliest stage of atherosclerosis, characterized by the impaired endothelium’s physiological vasoprotective function [26]. Fatty streak formation is the stage where subendothelial accumulation of lipid-laden macrophages/VSMCs known as foam cells [38] occurs, along with the recruitment of T cells and secretion of pro-inflammatory cytokines and chemokines, such as IL-1, IL-6, TNF-α, and monocyte chemoattractant protein-1 (MCP-1) [39] (Figure 1 and Table 1).

It is important to note that endothelial activation and dysfunction are distinct yet interconnected processes, both involving changes in EC behavior. Endothelial activation is typically an acute and reversible response to injury or inflammation, characterized by increased expression of adhesion molecules, cytokine release, and enhanced leukocyte recruitment. In contrast, endothelial dysfunction represents a chronic pathological state, marked by impaired vasodilation, oxidative stress, inflammation, and loss of barrier integrity [26]. Prolonged endothelial activation can lead to dysfunction, as the persistent inflammation and oxidative stress damage the endothelium and impair its physiological functions [66]. In pathologies like atherosclerosis and hypertension, the transition from endothelial activation to endothelial dysfunction is a critical event. Thus, endothelial activation is often viewed as an early and potentially reversible phase, whereas endothelial dysfunction reflects a more advanced and irreversible stage of endothelial damage.

### 2.3. Endothelial Senescence

Senescence is a state of permanent cell cycle arrest, where cells remain metabolically active but lose their ability to proliferate and function properly. It is characterized by an increased expression of senescence-associated markers, such as p16, p21, and p53, along with decreased expression of telomerase, which maintains the length and integrity of telomeres [67]. Further, endothelial senescence induces the secretion of pro-inflammatory and pro-fibrotic factors, contributing to chronic low-grade inflammation and vascular remodeling [68]. Accelerated by processes such as oxidative stress, DNA damage, cellular inflammation, and shear stress [67], this condition is implicated in the pathogenesis of age-related vascular diseases, such as atherosclerosis, aneurysm, and vascular calcification [67,68]. EC apoptosis and necrosis reduce endothelial integrity and promote exposure of the subendothelial matrix, which promotes plaque growth and instability. Similarly, oxidative stress, driven by the accumulation and generation of ROS, is responsible for cellular senescence, ultimately contributing to age-related endothelial dysfunction. While moderate levels of ROS play crucial roles in cellular functions, an excess of ROS induces detrimental effects, including DNA damage, which activates the p53-dependent pathway, leading to cell cycle arrest via p21 [69,70,71].

### 2.4. LDL Transcytosis Across the Arterial Endothelium

The LDL transport from plasma to the arterial sub-endothelial space is considered a rate-limiting step in atherogenesis and plays a critical role in determining the spatial distribution of atherosclerotic lesions. While the arterial endothelium is permeable to water and small molecules (<6 nm diameter), the transport of macromolecules, including LDL particles (22 to 28 nm), across the EC layer is tightly regulated. Due to the restrictive nature of intercellular junctions, including tight junctions and gap junctions, paracellular transport of LDL is nil to minimal [72]. Instead, LDL primarily crosses the endothelium via transcytosis, a transcellular process [73,74,75]. Emerging evidence supports the role of endothelial transcytosis in LDL transport via caveolae, flask-shaped invaginations of the plasma membrane [76]. Key proteins localized within caveolae, such as activin-like kinase 1 (ALK1) and scavenger receptor class B type 1 (SR-B1), have been shown to directly bind LDL and mediate its transcytosis. The molecular mechanisms governing this process have been recently reviewed in detail [76,77].

### 2.5. Endothelial-to-Mesenchymal Transition

Endothelial-to-mesenchymal transition (EndMT), a dynamic cellular phenomenon, is recognized to play important roles in several CVDs, including atherosclerosis, valvular disease, and pulmonary arterial hypertension [78,79]. During EndMT, ECs lose their intercellular junctions, degrade the basement membrane, and undergo phenotypic transformation to acquire mesenchymal characteristics [80]. This transition is driven by the induction of key EndMT-related transcription factors (Snai1, Snai2, Twist-1, Zeb1, and Zeb2), leading to the progressive loss of typical EC markers and the upregulation of mesenchymal markers vimentin, fibronectin, SM22α, etc. [79]. These phenotypic changes contribute to vascular inflammation, plaque development, and destabilization [81,82,83,84,85]. EndMT also plays a critical role in endothelial activation and dysfunction, promoting maladaptive tissue responses across various disease states [86]. Radiation therapy has been reported to induce EndMT in HAoECs, marked by increased expression of mesenchymal markers and decreased levels of endothelial markers (CD31, VE-cadherin). These changes were also observed in irradiated *Apoe^−^*^/*−*^ mice with elevated oxLDL, linking oxLDL-driven EndMT to radiation-induced atherosclerosis [87]. The involvement of EndMT in atherosclerosis has been comprehensively reviewed previously [80,88].

## 3. Mechanisms of EC Inflammation

### 3.1. Oxidative Stress in EC Inflammation

The imbalance between the ROS production and their scavenging is called oxidative stress [89], which promotes EC inflammation and dysfunction. This section briefly covers different types of ROS, reactive nitrogen species (RNS), their sources, and roles in endothelial health.

#### 3.1.1. Reactive Oxygen Species and Their Role in EC Inflammation

ROS comprise a group of molecules derived from molecular oxygen, which are highly reactive and capable of damaging cellular components and triggering inflammatory responses (Table 2). Vascular ECs produce ROS in response to various stimuli, such as shear stress, hyperglycemia, angiotensin II (Ang II), and cytokines. Multiple sources contribute to ROS production in ECs, including NADPH oxidases (NOXs), mitochondria, cytochrome P450, and xanthine oxidase.

**Table 2:** Common ROS and RNS molecules involved in EC inflammation [90].

Molecule	Function	Refs.
Superoxide anion (O_2_^•−^)	It is produced by the one-electron reduction of molecular oxygen (O_2_). Superoxide can activate various signaling pathways in ECs, such as MAPK, NF-κB, and nucleotide-binding domain, leucine-rich-containing family, pyrin domain–containing-3 (NLRP3), and induce the expression of pro-inflammatory cytokines, chemokines, and adhesion molecules.	[90,91]
Hydrogen peroxide (H_2_O_2_)	It is produced by the two-electron reduction of O_2_ or by the dismutation of superoxide. H_2_O_2_ can modulate the activity of various transcription factors in ECs, such as NF-κB, Nrf2, and AP-1, and regulate the expression of genes involved in inflammation, antioxidant defense, and cell death.	[90,92,93]
Hydroxyl radical (^•^OH)	It is the most reactive and damaging ROS molecule, which is produced by the one-electron reduction of H_2_O_2_ or Fenton reaction. Hydroxyl radical can cause oxidative damage to various biomolecules in ECs, such as lipids, proteins, and DNA, and trigger inflammation, apoptosis, and senescence.	[90,92]
NO radical (^•^NO)	NO is a free radical, which readily reacts with various molecules, particularly iron centers and oxygen. It plays critical roles in vasodilation, neuronal signaling, and microbial defense.	[94]
Peroxynitrite (ONOO^−^)	It is reactive nitrogen species, produced by the reaction of superoxide with NO. Peroxynitrite can impair the function of various enzymes and proteins in ECs, such as nitric oxide synthase (NOS), cyclooxygenase (COX), and SOD, and induce inflammation, nitrosative stress, and endothelial dysfunction.	[90,95]

Among these, flavocytochrome enzymes NOXs are the major ROS sources in ECs [96,97]. These enzymes catalyze the transfer of electrons from cytosolic NADPH to molecular oxygen, producing superoxide ion (O_2_^•−^) and hydrogen peroxide (H_2_O_2_). NOX-derived ROS serve several physiological roles, including host defense, biosynthesis, cellular signal transduction, and vascular function [98,99]. Mitochondria also produce ROS, but they are not the main source in ECs, as mitochondrial oxidative phosphorylation contributes minimally to ATP production in these cells [100,101]. Additional ROS sources in ECs include lysosomes, peroxisomes, and endoplasmic reticulum (ER) [102,103,104,105]. Various ROS types, their cellular sources, and functions in vascular health have been comprehensively reviewed previously [106,107]. In humans, 7 NOX isoforms—NOX1, NOX2, NOX3, NOX4, NOX5, Duox1, and Doux2 have been identified, while mice and rats lack NOX5 [108]. These NOX isoforms are localized in various subcellular locations, such as the plasma membrane, perinuclear membrane, and ER [97,98,99,109]. Four members of NOX family, including NOX1, NOX2, NOX4, and NOX5, are important sources of ROS in the vasculature [110]. NOX1, NOX2, and NOX5 produce O_2_^•−^ in response to stimuli like shear stress, growth factors, and inflammatory cytokines, while NOX4 generates H_2_O_2_. ROS derived from NOX1, NOX2, and NOX5 have been shown to induce EC apoptosis, inflammation, and endothelial dysfunction [100,111]. Additionally, NOX1- and NOX4-derived ROS contribute to cell cycle arrest in the S phase and induce EC senescence, which are the features of EC activation [106].

NOX2 produces O_2_^•−^ both intracellularly and extracellularly. Intracellular O_2_^•−^ is neutralized by cytoplasmic superoxide dismutase (SOD1), while extracellular O_2_^•−^ is scavenged by SOD3, converting it to H_2_O_2_. Superoxide radicals produced by NOX2 in the endothelium also react with NO, reducing its bioavailability and impairing endothelium-dependent vasorelaxation [112]. In isolated aortic tissue, the extent of endothelium-dependent relaxation is inversely correlated with NOX2 expression [113]. There is a strong interplay between NOX-derived ROS and NO signaling. For instance, ECs exposed to high glucose levels display increased expression and activity of NOX2, which is negatively correlated with endothelial nitric oxide synthase (eNOS) expression and NO production [114]. Conversely, treatment with NO donors downregulates NOX2 expression and O_2_^•−^ generation [115]. Further, oscillatory shear stress (OSS) has been demonstrated to induce NOX2 expression, promote O_2_^•−^ generation, and stimulate monocyte adhesion [116]. Moreover, NOX2-produced ROS in human microvascular ECs (MVECs) promote cell cycle arrest and apoptosis by increasing expression of cyclin-dependent kinase inhibitor, p21Cip1 and cell cycle regulatory protein, p53 [117].

NOX4 is the most abundantly expressed NOX isoform in the cardiovascular system [118]. Its expression in ECs is regulated by mechanical stimuli—downregulated by laminar shear stress and upregulated by OSS [119,120]. NOX4 plays a key role in EC proliferation and cell cycle progression [121]. Specifically, NOX4 knockdown reduces epidermal growth factor-induced proliferation, whereas its overexpression promotes EC growth [121,122]. The role of NOX4 in apoptosis appears context-dependent. In MVECs NOX4 overexpression protects against serum starvation-induced apoptosis, while it mediates apoptosis in response to TNF-α stimulation [121,123,124]. Similarly, in various cell types and tissues, NOX4-induced senescence contributes to the development of CVDs [125,126]. Notably, NOX4 expression increases with age, particularly in the aorta, renal cortex, and medulla [113,127,128]. The functional versatility of NOX4 is highlighted by its capacity to respond to diverse stimuli and its involvement in multiple cellular processes, including differentiation [129,130], migration [131,132], proliferation [133], apoptosis [124,134], senescence [126], and inflammation [135,136]. However, further research is required to completely determine if NOX4 has similar functions in the cardiovascular system.

ROS play a pivotal role in the pathogenesis of atherosclerosis via regulating various signaling pathways in ECs. Figure 1 summarizes ROS-stimulated signaling pathways, including MAPK, NF-κB, Nrf2, and NLRP3 cascades. Several in vivo studies have reported that different NOX isoforms promote atherosclerosis [122,137,138]. NOX1 expression is increased in diabetic atherosclerotic mice [139], and its deletion in diabetic mice suppresses lesion formation, ROS generation, leukocyte adherence, and macrophage infiltration [140,141]. In contrast, EC-specific NOX2 overexpression does not affect atherosclerosis development in the aortic root or descending aorta [142]. Interestingly, NOX4 deficiency in *Apoe^−^*^/*−*^ mice leads to reduced H_2_O_2_ generation and increased atherosclerotic lesion formation [143]. Moreover, patients with symptomatic carotid artery stenosis exhibit reduced NOX4 mRNA and H_2_O_2_ levels, suggesting a protective role of NOX4 in plaque stabilization [144]. These results indicate that NOX4 may exert atheroprotective effects, potentially through H_2_O_2_-mediated upregulation of eNOS. The absence of NOX5 in rodents has limited investigations into its role in the development of atherosclerosis. However, studies using EC-specific NOX5 knock-in mice indicate that its expression does not promote plaque formation [145]. This information suggests the complex and isoform-specific roles of NOX-derived ROS in endothelial biology and atherosclerosis.

#### 3.1.2. Sources of NO and Endothelial Cell Biology

NO is synthesized endogenously by three isoforms of NOS, which catalyze the NADPH-dependent oxidation of L-arginine to L-citrulline and NO. These enzymes are expressed in various vascular and immune cell types [146]. The NOS family includes two constitutive isoforms—neuronal NOS (nNOS or NOS1) and eNOS (NOS3), as well as an inducible isoform (iNOS or NOS2). NO is a freely diffusible radical that readily crosses plasma membranes. It plays essential roles in neurotransmission, immunological defense, and maintenance of vascular tone [97]. The nNOS is a calcium-dependent enzyme predominantly found in the nervous system, gastrointestinal tract, and skeletal muscle. In contrast, iNOS is primarily expressed in activated immune cells, including macrophages and neutrophils, which generate a large amount of NO during inflammatory responses [97,147,148]. NO produced by eNOS promotes vasodilation by relaxing VSMCs, thereby reducing blood pressure. NOS enzyme activity requires cofactor tetrahydrobiopterin (BH4), and loss or oxidation of BH4 to dihydrobiopterin (BH2) leads to NOS uncoupling, resulting in the production of O_2_^•−^ instead of NO. Uncoupling of eNOS reduces NO bioavailability and promotes EC dysfunction. NO derived from eNOS is atheroprotective as it inhibits LDL oxidation, leukocyte adhesion, VSMC proliferation, and platelet aggregation [147,149]. In contrast, excessive NO generated by iNOS contributes to oxidative stress and atherogenesis through the formation of peroxynitrite and further depletion of BH4, which exacerbates eNOS uncoupling [150]. Interestingly, nNOS is also found in blood vessels, and may facilitate vasodilation, and exert anti-atherogenic effects [151].

#### 3.1.3. Effects of Various Circulating Risk Factors of Atherosclerosis on ROS Generation

Circulating risk factors for atherosclerosis, including oxidized LDL (oxLDL), high blood glucose levels, elevated free fatty acids (FFAs), Ang II, and pro-inflammatory cytokines, significantly stimulate ROS production by ECs, leading to endothelial dysfunction, and atherogenesis. These risk factors activate redox-sensitive pathways and impair NO bioavailability, which is a hallmark of vascular homeostasis disruption. OxLDL binds to different scavenger receptors (SR-B1, LOX-1, and CD36) present on the cell surface and augments intracellular ROS production specifically via NOX2 and NOX4 activation [152]. Concurrently, oXLDL suppresses eNOS expression and activation, consequently reducing NO release [153]. The increased ROS generation further causes oxidative modification of lipids and proteins, upregulation of adhesion molecules (VCAM-1, ICAM-1), and recruitment of monocytes, all of which contribute to EC inflammation and dysfunction [154]. High blood glucose levels (hyperglycemia) observed in diabetic individuals, promote ROS generation by multiple signaling pathways, which stimulate NOX enzymes and mitochondrial respiratory chain complexes, resulting in elevated ROS production and reduced NO bioavailability [155]. Moreover, elevated levels of circulating free fatty acids and Ang II also induce ROS production in ECs, leading to mitochondrial dysfunction and cellular inflammation [156,157]. Similar to the other circulating risk factors of atherosclerosis, various pro-inflammatory cytokines such as TNF-α, IL-6, and IL-1β upregulate NOX expression and induce ROS production. Further, these cytokines impair endothelial integrity by disrupting endothelial tight junctions, promote leukocyte adhesion, and decrease antioxidant capacity, thus facilitating atherosclerotic plaque development [158]. In summary, circulating atherogenic stimuli synergistically induce ROS generation in ECs, primarily via NOX activation and mitochondrial dysfunction.

### 3.2. Disturbed Flow in EC Inflammation and Atherosclerosis

The EC layer of blood vessels is exposed to varying magnitudes and patterns of shear stress exerted by blood flow, depending on blood viscosity and the vascular geometry. Shear stress alters EC structure and function, including cell orientation, cytoskeletal reorganization, permeability to macromolecules, and leukocyte attachment [159]. Unidirectional laminar blood flow, typically found in the straight region of arteries, is atheroprotective and shown to stimulate the secretion of potent vasodilators—NO and prostacyclin, while downregulating inflammatory cellular responses. In contrast, OSS caused by disturbed blood flow at branching points and curvatures triggers endothelial inflammation and contributes to plaque formation [160,161]. Therefore, atherosclerotic lesions are mainly detected in regions exposed to disturbed flow, including the inner curvature of the aortic arch, carotid bifurcations, branch points of the coronary, and infrarenal arteries, etc. Due to exposure to disturbed flow, ECs in these arterial regions exhibit reduced production of anti-inflammatory and vasodilatory NO, along with increased expression of adhesion molecules (ICAM-1, VCAM-1, and E-selectin), facilitating leukocyte attachment and transmigration across the intima [160,162,163].

Shear stress activates various EC mechanosensors, such as integrins, tyrosine kinase receptors (e.g., VEGFR-2), and G-protein coupled receptors. Integrins mediate the effects of shear stress on EC cytoskeleton by activating MAPKs and focal adhesion-associated kinases [164,165]. VEGFR-2 activation by shear stress promotes downstream AKT phosphorylation via various proteins, including VE-cadherin, β-catenin, and PI3K [166,167]. These signaling pathways induce EC inflammation, proliferation, and apoptosis. A key mechanism by which OSS promotes EC inflammation is the impaired expression of eNOS, the principal enzyme responsible for NO production in ECs. Another important regulator of vascular health is Krüppel-like factor 2 (KLF2), a transcription factor that is highly expressed in ECs and induces eNOS expression. Laminar blood flow, but not atheroprone-OSS, upregulates KLF2 mRNA levels, leading to increased eNOS expression and NO production [168]. Other transcription factors, including PKA [169], Nrf2 [170], SIRT1 [171], and HDACs (e.g., HDAC5) [172], also contribute to laminar shear stress-mediated activation of eNOS. Furthermore, in vivo studies have shown that disturbed flow promotes EC proliferation in the early stages of atherogenesis. Interestingly, prolonged laminar flow in vitro reduces EC proliferation and leads to cell cycle arrest in the G0/G1 phase, whereas OSS accelerates EC turnover and the G0/G1 to S transition [173,174]. Thus, the vascular EC layer, through its response to hemodynamic forces, serves as a critical regulator of vascular physiology and pathobiology in both health and disease. The role of disturbed flow in regulating vascular dysfunction has been nicely reviewed previously [160,175].

### 3.3. ER Stress in EC Inflammation and Atherosclerosis

The ER plays important roles in protein synthesis, folding, trafficking, lipid synthesis, and calcium homeostasis. However, the ER can become dysfunctional due to the accumulation of unfolded or misfolded proteins in its lumen [176,177]. During ER stress, unfolded protein response (UPR) is induced as a compensatory mechanism to restore ER homeostasis by enhancing protein folding, degrading misfolded proteins, and reducing protein synthesis [178]. However, under the setting of persistent ER stress, the UPR stimulates apoptosis, inflammation, and oxidative stress, which can further damage cells, and has been shown to promote atherosclerosis [178,179]. ER stress activates various signaling pathways in ECs, such as MAPK, NF-κB, Nrf2, and NLRP3, which modulate the expression of genes involved in inflammation, antioxidant defense, and cell death [180].

Exposure of ECs to various sources of ER stress, such as elevated cholesterol levels, ROS, calcium imbalance, and shear stress, can impair normal cellular functions, including vasodilation, anticoagulation, anti-inflammation, and anti-oxidation [181,182]. For instance, ECs exposed to oxidized phospholipids undergo ER stress response and express higher levels of adhesion molecule, VCAM-1 [183]. Within human atherosclerotic lesions, endothelial regions rich in oxidized phospholipids exhibit increased ER stress markers, which promote the initiation and progression of plaque formation [184]. Throughout the course of atherogenesis, modified LDL, characterized by modifications, including oxidation, glycosylation, or phospholipolysis, perturbs calcium homeostasis within the ER, triggering UPR in ECs and promoting oxidative stress [185]. Earlier investigations have shown that phospholipolyzed LDL elicits inflammatory responses in ECs via ER stress pathways [181,186,187]. Furthermore, studies have demonstrated that oxLDL induces inflammatory pathways in ECs, which are responsible for EC damage through the activation of inflammasome-mediated apoptosis signal-regulating kinase 1 (ASK1) and NLRP3 [186,188]. In summary, modified LDL exerts significant regulatory effects on ER stress-mediated endothelial dysfunction, inflammation, and apoptosis within atherosclerotic vessels [179,187,188].

Under ER stress, an imbalance of the Bcl-2 family, mediated by CHOP, activates proapoptotic proteins on the mitochondrial membrane, leading to the release of cytochrome c and subsequent mitochondrial-dependent apoptosis [189]. This process, combined with disrupted calcium homeostasis, results in diminished mitochondrial function and elevated levels of NADPH and ROS in ECs within the pathological milieu of atherosclerosis [190,191]. Moreover, ER stress induces cytosolic calcium overload, triggering the activation of procaspase-12 into caspase-12 on the ER membrane of ECs. Consequently, caspase-3 and apoptosis are activated in these cells, alongside calpain-mediated activation of caspase-9 [179]. In short, dysfunctional ER responses, triggered by factors like modified LDL and oxidative stress, lead to inflammation, apoptosis, and oxidative damage to ECs and exacerbate atherosclerotic plaque formation and progression.

### 3.4. Autophagy in EC Inflammation and Atherosclerosis

Autophagy is a catabolic cellular process that degrades and recycles damaged or unwanted cellular components, such as proteins, organelles, and lipids [192,193]. Autophagy serves as both a metabolic process for degrading organelles and intracellular substances and as a stress response to conditions like starvation and oxidative stress. It plays a crucial role in cell proliferation, differentiation, and aging. The regulation of autophagy involves several signaling pathways, among which the mammalian target of rapamycin complex 1/unc-51-like autophagy activating kinase 1 pathway and the silent mating type information regulation 2 homolog 1/Forkhead box protein O1 (Sirt1/FoxO1) pathway are relevant to ECs [194,195,196]. Sirt1 expression in vascular ECs promotes angiogenesis and exerts anti-inflammatory and anti-atherosclerotic effects by regulating transcription factors such as FoxO1 and NF-κB, as well as eNOS activation [197,198,199]. FoxO1 is also intricately linked to autophagy regulation, as it modulates the expression of key autophagy-related proteins like microtubule-associated protein 1 light chain 3, autophagy-related protein 5 (Atg5), and Beclin-1 [196,200,201].

Autophagy has both anti-atherogenic and pro-atherogenic effects, depending on the type, intensity, and duration of the stimulus, and the stage of atherosclerosis [202]. Numerous stimuli can activate autophagy within atherosclerotic plaques, including ROS, LDL, TNF-α, and other inflammatory factors [203,204]. Autophagy can reduce atherogenesis and help maintain a stable plaque phenotype. Inhibition of EC’s autophagy induces the expression and secretion of various factors VCAM-1, ICAM-1, E-selectin, MCP-1, and IL-8, which facilitate the recruitment and activation of leukocytes and promote foam cell formation, thereby increasing the risk of arterial thrombosis [202]. Additionally, Perrotta et al. demonstrated that pharmacological inhibition of glycolytic flux with a small molecule inhibitor 3-[3-pyridinyl]-1-[4-pyridinyl]-2-propen-1-one (3PO) induces autophagy and impairs NF-κB signaling, resulting in the inhibition of TNF-α-mediated upregulation of VCAM-1 and ICAM-1 [205]. In instances of oxidative stress, damaged mitochondrial DNA (mtDNA) evades autophagic clearance and triggers a potent inflammatory response in the arterial wall [206]. Enhanced autophagy significantly reduces chronic vascular inflammation and suppresses atherosclerosis. Conversely, inhibition or impairment of autophagy exacerbates the inflammatory response [202,207]. These findings suggest that autophagy protects against endothelial inflammation. Transient knockdown of the essential autophagy gene ATG7 results in increased intracellular levels of both intermediate-density lipoprotein and oxLDL, suggesting that in ECs, autophagy serves as an important mechanism for regulating excess exogenous lipids [208,209]. All these studies demonstrated that endothelial autophagy has a potential role in EC inflammation-dependent atherosclerosis. Therefore, a better understanding of the molecular mechanisms regulating autophagy in ECs may be essential in developing novel and effective strategies to prevent EC dysfunction/inflammation.

## 4. Matricellular Proteins in EC Inflammation and Atherosclerosis

Matricellular proteins are a group of non-structural proteins that modulate various cellular functions, such as adhesion, migration, proliferation, differentiation, and survival, by interacting with different molecules, including cell surface receptors, proteases, hormones, and ECM components. They are often highly expressed during embryonic development, repair, and remodeling, and regulate inflammation and immune responses. Remarkably, these proteins exhibit both pro-inflammatory and anti-inflammatory effects, depending on the context and receptors they interact [11,210]. Therefore, targeting these proteins or their receptors may offer new therapeutic strategies for modulating inflammation and associated pathologies. These proteins are characterized by distinct domains that possess enzymatic activity capable of modifying ECM components or regulating the activities of various growth factors. By serving as molecular bridges, these proteins facilitate communication and crosstalk among growth factors, proteases, cytokines, and other macromolecules within the cellular microenvironment [211]. The matricellular protein family includes a diverse range of molecules, including thrombospondins (TSPs), CCN proteins (CYR61, CTGF, NOV), SPARC (secreted protein acidic and rich in cysteine), osteopontin, tenascins, Rspondins and others [11]. Each member of this protein family exhibits unique structural features and functional properties, contributing to the dynamic regulation of cellular and tissue homeostasis. Furthermore, their dysregulated expression and activity have been implicated in various pathological conditions, including cancer, fibrosis, CVDs, and inflammatory disorders [212,213]. Here, we summarize the role of various matricellular proteins in EC inflammation and atherosclerosis (Table 3).

**Table 3:** Role of matricellular proteins in regulating vascular phenotype.

Protein	Primary Receptors	Signaling Pathway and Phenotype	Cell/Animal/Human Model Type	Role	Refs.
TSP1	CD47 CD36	Decreases cAMP/cGMP levels by inhibiting EC NO production	Bovine aortic ECs Human umbilical vein ECs *Thbs1*^−/−^ and *Cd47*^−/−^ mice arteries	EC dysfunction and pro-hypertensive	[214]
		*Thbs1* deletion prevents leptin-induced atherosclerosis Deletion blocks leptin-induced vascular inflammation Deletion inhibits SMC dedifferentiation	*Apoe*^−/−^ and *Apoe*^−/−^*/Thbs1*^−/−^ mice	Pro-atherogenic Pro-inflammatory Pro-atherogenic	[215]
		In the early stage, a deficiency of *Thbs1* reduces plaque area In the advanced stage, *Thbs1* loss promotes plaque necrosis	*Apoe*^−/−^ and *Apoe*^−/−^*/Thbs1*^−/−^ mice	Pro-atherogenic Anti-plaque vulnerability	[216]
		*Thbs1* deletion in mice promotes maladaptive remodeling in response to pressure overload via inhibiting Thbs1/integrin β1/YAP signaling *Thbs1* deletion inhibits neointima formation upon carotid artery ligation	*Thbs1*^−/−^ mice	Promotes intimal hyperplasia	[217]
COMP	α5β1 α7β1	Lack of *Comp* induces aging-related vascular dysfunction, stiffness, and senescence	*Comp*^−/−^ mice	Promotes vascular function	[218]
		*Comp* deletion augments atherosclerosis	*Apoe*^−/−^ and *Comp*^−/−^/*Apoe*^−/−^ mice	Anti-atherogenic	[219]
RSPO2	LGR4	Suppresses lymphangiogenesis via PI3K-AKT-eNOS signaling and inhibits Wnt-β-catenin pathway in lymphatic ECs	Human dermal lymphatic ECs	Anti-lymphangiogenic Pro-atherogenic	[220]
		Perivascular application of LGR4-ECD promotes arterial lymphangiogenesis and reduces atherosclerosis	*Apoe*^−/−^ mice		
RSPO1	LGR4-5	Wnt/β-catenin/VEGFaa-induced abnormal angiogenesis	Zebrafish	Pro-angiogenic	[221]
RSPO3	LGR4-5	Non-canonical WNT/Ca^2+^/NFAT signaling and vascular defects	EC-specific *Rspo3*-deficient mice	Pro-angiogenic	[222]
Tenascin-C	Integrins	TN-C polymorphisms correlate with atherosclerosis/CAD	Human aorta samples and CATHGEN cardiovascular study	Three SNPs correlate with atherosclerosis	[223]
Osteopontin (OPN/SSP1)	Integrins CD44	Deletion reduces atherosclerosis Deletion stimulates vascular calcification Deficiency reduces atherogenesis	*Apo*^−/−^/*Spp1*^−/−^ mice *Apoe*^−/−^*/Ldlr*^−/−^ /*Spp1*^−/−^ triple knockout mice	Pro-atherogenic Pro-atherogenic	[224]

		Expression levels associate with plaque severity	Human aorta samples	Pro-atherogenic	[225]
CCN1	Integrins	Upregulated levels in atherosclerotic aortas of *Apoe*^−/−^ mice Promotes atherosclerosis	*Apoe*^−/−^ mice	Pro-atherogenic	[226]
		Elevated Ccn1 expression in atherosclerotic arteries	*Apoe*^−/−^ mice and human ECs	Mediates TNFα-induced EC apoptosis	[227]
		Promotes neovascularization	C57BL/6 wild-type mice and human venous ECs	Pro-angiogenic	[228]

### 4.1. Thrombospondins

There are five different types of thrombospondins, named TSP1 to TSP5, based on their molecular structures, functions, and oligomerization status, which are encoded by five distinct genes, namely *THBS*1 to 5 [229]. Among them, TSP1 and TSP2 are extensively studied and play significant roles in various biological processes. They contribute to inflammatory responses, regulate angiogenesis during tumor growth, and promote EC apoptosis. TSP1 and TSP2 via binding to various receptors found on ECs, including CD36 [230], CD47 [231], LRP-1 [232], and integrins (α9β1, α6β1, αvβ3, and αIIββ3) [233,234,235,236] regulate several signaling pathways critical for cellular function [11,237]. TSP2 has been demonstrated to inhibit the expression of vascular endothelial growth factor A (VEGF-A), a potent angiogenic factor. Via blocking binding of VEGF to its receptors on ECs, TSP2 prevents VEGF-VEGFR-mediated angiogenic signaling [238]. In addition, TSP2 exerts inhibitory effects on the activities of MMP-2 and MMP-9, enzymes involved in angiogenesis and inflammation [239]. Further, TSP2 activates the tissue inhibitor of metalloproteinases 3 (TIMP-3), an inhibitor of MMPs and other proteases. While TSP3, TSP4, and TSP5 are less characterized and involved in tissue development, repair, and remodeling. Notably, TSP4 via CD44 activation modulates the ECM composition and the mechanical properties of atherosclerotic plaques by interacting with collagen and elastin [240], and affects plaque vulnerability and rupture. Additionally, Frolova et al., reported that TSP4 triggers inflammatory signaling in ECs and monocytes/macrophages, promoting the accumulation of macrophages in atherosclerotic plaques [241]. TSP5, also known as cartilage oligomeric matrix protein (COMP), is mainly expressed in cartilage and modulates the differentiation and survival of chondrocytes [242]. TSP5 functions by interacting with integrins (α7β1, αvβ3, α5β1, α5β3) as well as CD47 [11,243,244].

#### 4.1.1. Thrombospondin1 (TSP1)

TSP1 plays an important role in various biological processes, including angiogenesis [245], lymphangiogenesis [246], inflammation [247], and atherosclerosis development [215,216,246]. TSP1 has been shown to bind and activate latent TGF-β, thereby modulating TGF-β-stimulated signaling in various diseases, including diabetes, liver fibrosis, pulmonary arterial hypertension, arterial stiffening, cardiomyopathy, and tumors [248]. Antibody-mediated blockade of TSP1-induced signaling has been shown to promote re-endothelialization and suppress neointima formation in the carotid arteries of rats after balloon injury, indicating the detrimental role of TSP1-stimulated signaling in neointima formation [249]. Moreover, global *Thbs1* deletion in mice on *Apoe^−^*^/*−*^ background led to reduced plaque area compared with control *Apoe*^−/−^ mice following leptin stimulation [215]. In contrast, global *Apoe*^−/−^/*Thbs1*^−/−^ double knockout mice exhibited augmented plaque maturation; however, this effect was observed only during the advanced stage of atherosclerosis [216]. In early atherogenesis, *Thbs1* loss reduced lesion formation; however, in the later stages, its deficiency was associated with increased inflammation, impaired phagocytosis, and elevated ECM remodeling [216]. Despite these findings, the impact of EC-specific *Thbs1* deletion or overexpression on atherosclerotic lesion formation remains unexplored.

The effects of TSP1 on EC biology have been extensively studied in vitro. Activation of CD36 by TSP1 initiates a cascade involving Fyn, JNK, and p38/MAPK, ultimately triggering apoptosis via the activation of caspase-8 and caspase-9-dependent pathways. Additionally, TSP1 via CD36 activation triggers caspase-dependent cell death mechanisms and ultimately induces EC apoptosis [250,251,252]. Consistently, *CD36*-silencing in human umbilical vein ECs (HUVECs) counteracts the inhibitory effects of TSP1 on migration and tube formation, indicating the role of TSP1-CD36 signaling axis in angiogenesis [253]. Further, in vitro studies demonstrated that TSP1 and stimulation of CD36 receptor with a CD36 agonist antibody inhibit NO-stimulated EC responses, including chemotaxis, adhesion, and proliferation [254], suggesting an antagonism between the TSP1-CD36 axis and proangiogenic signaling downstream of NO. Besides, *CD36* knockdown in MVECs or genetic *Cd36* deletion in mice revealed that TSR (CD36-binding domain of TSP1)-induced SHP-1/VEGFR2 complex formation is mediated by CD36 both in vitro and in vivo, and silencing of *SHP-1* in MVECs abrogates TSR-mediated inhibition of VEGFR2 phosphorylation [255]. On the other hand, TSP1-mediated suppression of NO signaling remains unaltered in CD36-null ECs; however, it is reduced in CD47-deficient ECs, demonstrating the essential role of CD47 in this process [256]. Further, TSP1 by binding to CD47 in ECs inhibits eNOS, leading to reduced NO production and bioavailability [257]. Consistently, TSP1 treatment has been demonstrated to promote VCAM-1 and ICAM-1 expression and increase monocyte adhesion to human ECs [258]. These observations point out the significant role of TSP1-induced signaling in EC inflammation. Recently, Peng et al. reported elevated levels of TSP1 in the peripheral blood of pediatric patients with pulmonary hypertension, and suppression of Sugen/hypoxia-induced pulmonary hypertension and EndMT with pharmacological inhibition of TSP1 [259]. Additionally, silencing of *THBS1* in human pulmonary ECs inhibited hypoxia-stimulated downregulation of EC markers, further supporting the role of TSP1 in hypoxia-induced EndMT [259]. Moreover, increased TSP1 expression has been associated with EndMT in a mouse model of complete left carotid artery ligation, indicating the potential role of TSP1 in EndMT-induced atherosclerotic lesion formation [260].

In addition, TSP1-CD47 signaling regulates thrombosis/hemostasis [261], immune responses, and mitochondrial function in various cell types [262,263]. Moreover, TSP1-CD47 signaling axis is involved in the regulation of senescence, self-renewal, and inflammation. Experimental data from CD47-deficient ECs and muscle explants from global *Cd47* knockout mice revealed that TSP1 requires CD47 to inhibit NO signaling [256]. CD47 deficiency in ECs downregulates inflammatory cytokines and chemokines, including TSP1 [264,265]. Additionally, a recent investigation by Singh et al. reported that the deletion of *Cd47* specifically in ECs attenuates atherosclerotic lesion formation in mice [266]. Further, the authors observed increased internalization of apoptotic cells (efferocytosis) by *Cd47*-deficient ECs compared with control cells possibly via upregulating expression of efferocytosis receptor FasL, CX3CL1, Gpr132, MerTK, Scarb1, Sirpα, TSP1, and HMGB1 [266]. In addition to this, the expression of CD47 on non-tumor cells, like ECs, is crucial in regulating tumor angiogenesis. Particularly, the absence of CD47 expression on ECs markedly boosts angiogenesis, thereby effectively mitigating hypoxia-induced tumor necrosis and expediting tumor progression [265]. This suggests that targeting CD47 and/or TSP1 can be a promising therapeutic strategy to delay EC senescence and mitigate inflammation.

**Figure 2:**
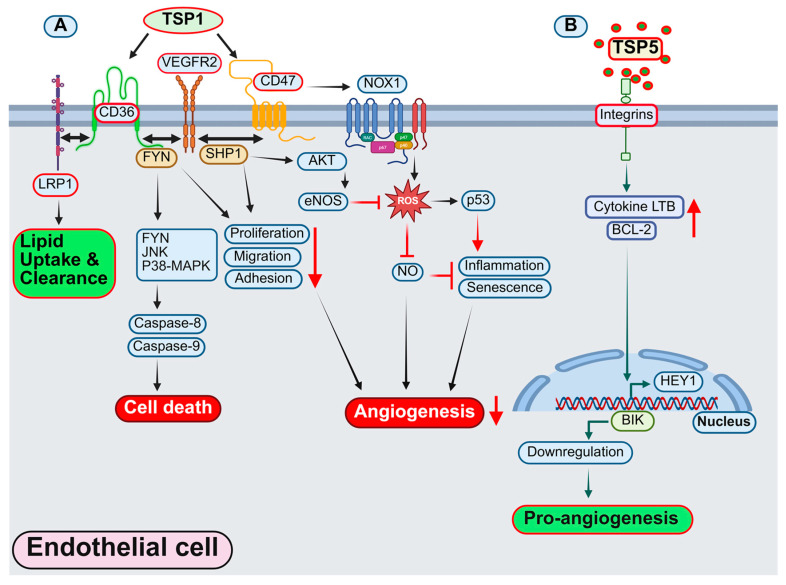
Role of TSP1 and TSP5 in endothelial dysfunction: Schematic representation of various receptors present on ECs that interact with matricellular proteins TSP1 and TSP5 and downstream signaling pathways. (**A**) TSP1 via binding to CD36 facilitates the formation of a CD36-Fyn complex. This complex amplifies Fyn-mediated signal transduction. Additionally, TSP1 induces the dissociation of VEGFR2 from CD47, leading to the association of VEGFR2 with CD36. (**B**) The illustration of interactions between TSP5 and integrins and downstream key signaling pathways. TSP5-integrin complex modulates the expression of cytokine LTB, BCL2, HEY1, and BIK, ultimately promoting angiogenic responses. ↑ (red): increased signaling/expression; ↓ (red): increased signaling/expression; ↔ (black): interaction; Ⱶ (red): inhibition.

TSP1 induces ROS generation in ECs, leading to oxidative stress and ultimately inducing cell senescence. TSP1-mediated Nox1 activation in human pulmonary artery ECs increases ROS production and elevates levels of transcription factor p53, promoting EC senescence [267]. Importantly, blocking of CD47 receptor with an antibody prevents TSP1-induced ROS generation [267], suggesting TSP1 promotes senescence, inflammation, and apoptosis in ECs by increasing ROS generation, inhibiting NO signaling, and/or decreasing antioxidant levels [245,268,269]. Various in vivo studies have explored the role of TSP1 in EC ROS generation and its implications in metabolic and CVDs. Mice deficient in TSP1 exhibit reduced ROS production, decreased p21 expression, attenuated p53 activity, and diminished aging-induced senescence in lung tissue compared with wild-type mice [267] (Figure 2A). Furthermore, TSP1-induced CD47 activation stimulates ROS generation in human pulmonary artery ECs, and this signaling pathway emerged as a pivotal player in sickle cell-associated vasculopathy, contributing to the development of pulmonary hypertension [270,271,272]. Recent research by our group demonstrated that TSP1 via CD47 activation in lymphatic ECs (LECs) suppresses lymphangiogenesis by inhibiting AKT-eNOS signaling and inducing ROS production [246]. Further, deletion of CD47 specifically in LECs augmented lymphangiogenesis and attenuated atherosclerosis in hypercholesterolemic mice [246] (Figure 2A). In another type of vascular cell, VSMCs, TSP1 stimulates Nox1-mediated superoxide production as determined using cytochrome c reduction and electron paramagnetic resonance assays [273]. Further, the inhibition of CD47-mediated signaling employing blocking antibody and gene silencing abrogates TSP1-induced ROS production in vitro and ex vivo. This increased ROS production, in turn, disrupts redox signaling within the vasculature, consequently impairing VSMC-dependent vasorelaxation, a crucial mechanism for maintaining vascular tone and blood pressure (BP) regulation [273]. Such vascular dysfunction contributes to pathological conditions like hypertension and atherosclerosis, highlighting the critical role of the TSP1-CD47 axis in vascular homeostasis and disease progression. Additionally, in macrophages, TSP1-stimulated Nox1 activation triggers the dephosphorylation of the actin-binding protein cofilin [274]. This process subsequently induces cytoskeletal rearrangement, which is associated with increased LDL uptake by macrophages via stimulating macropinocytosis, thus promoting atherosclerosis progression [274,275]. Altogether, these findings suggest that inhibition of TSP1-mediated signaling may be beneficial for EC function, vascular tone, and homeostasis.

#### 4.1.2. Thrombospondin 5/Cartilage Oligomeric Matrix Protein (COMP)

TSP5/COMP functions by binding to α5β1 and is mainly expressed in cartilage, bone, and tendon, where it regulates the development and maintenance of the ECM [244]. Expression of COMP has also been detected in both inflammatory and fibrous atherosclerotic plaques in mice [219]. In a recent study, Hultman et al. associated arterial COMP expression with symptomatic carotid atherosclerosis [276]. Additionally, COMP levels were positively correlated with plaque lipid content and CD68-positive areas but inversely correlated with lesional collagen content, suggesting a link between COMP expression and plaque vulnerability. Another study reported higher circulating levels of COMP in patients with CAD and found a positive correlation between COMP levels and coronary artery calcium scores, advocating its potential as a biomarker for coronary artery calcification [277]. Interestingly, atheroprone *Apoe^−^*^/*−*^ mice lacking *Comp* exhibit increased plaque formation and vascular calcification [278]. Further, the authors of this study demonstrated that *Comp* deletion promotes atherosclerotic calcification by shifting macrophage phenotype toward pro-atherogenic and osteogenic types. Supporting this, Bond et al. reported larger plaques in brachiocephalic arteries of *Comp*-deficient *Apoe^−^*^/*−*^ mice [219]. Moreover, COMP suppresses disturbed flow-induced EC activation by interacting with integrin α5, thereby maintaining endothelial homeostasis. Increased EC activation was observed in *Comp*^−/−^ mice, particularly in the aortic arch under normal conditions and following partial carotid artery ligation [279]. Notably, treatment with a COMP-derived peptidomimetic (CCPep24) that mimics COMP’s interaction with integrin α5 reduced endothelial activation and atherogenesis in vivo [279], indicating the protective role of COMP in EC inflammation and atherosclerosis.

Various studies have investigated TSP5 expression and function in vascular cells and angiogenesis [280,281]. TSP5 has been shown to have pro-angiogenic activity, potentially via inducing the expression of a proangiogenic gene *HEY1*, cytokine LTB, and antiapoptotic BCL2, while downregulating a proapoptotic gene, BIK [281] (Figure 2B). TSP5 enhances endothelium-dependent relaxation via regulating Piezo1/CaKMII/eNOS signaling pathway, thereby playing a protective role in BP regulation [282]. Moreover, overexpression of COMP in VSMCs reduces systolic and mean arterial BP during AngII-induced hypertension and promotes CaKMII/eNOS activation in arteries, suggesting the protective role of VSMC-derived COMP in BP regulation via NO release in ECs [282]. Similarly, COMP maintains endothelial homeostasis by attenuating disturbed flow-induced endothelial activation through its interaction with integrin α5 [279]. In both normal and pathological conditions, such as partially ligated carotid arteries in mouse models, global *Comp*-deficient mice exhibited increased aortic EC activation compared with control mice. Additionally, peptidomimetics derived from the C-terminal of COMP (CCPep24), which interacts with integrin α5, protect against EC activation and atherogenesis in vivo [279].

COMP also plays a pivotal role in the maintenance of VSMC contractile phenotype and confers protection against injury-induced neointima formation in Sprague-Dawley rats by interacting with α7β1 integrin [243]. Riessen et al. reported COMP expression in human VSMCs, human normal arteries, and those affected by atherosclerosis and restenosis [280]. In vitro experiments demonstrated that VSMCs strongly adhere to COMP-coated surfaces, facilitating their migration [280]. These findings suggest a potential role for COMP in vasculogenesis and vascular diseases such as atherosclerosis and restenosis by regulating VSMC migration and adhesion. Several studies have reported elevated COMP expression in fibrotic conditions through activation of skin fibroblasts and lung epithelial cells by TGF-β. TGF-β induces the conversion of fibroblasts and lung epithelial cells into myofibroblasts, leading to increased ECM synthesis and fibrosis. Upregulation of COMP expression enhances TGF-β signaling, establishing a positive feedback loop in idiopathic pulmonary fibrosis and fibrotic skin [283,284,285,286]. On the other hand, evidence from multiple mouse models indicates endothelial dysfunction and EndMT in vascular calcification [84,287,288]. Therefore, it is tempting to speculate a possible association of arterial COMP expression with TGF-β-dependent signaling in EC inflammation responsible for EndMT, atherosclerosis, and vascular calcification.

### 4.2. Osteopontin (OPN)

Osteopontin is a multifunctional glycoprotein, which plays a crucial role in inflammatory processes. It is also called secreted phosphoprotein 1 (SPP1). It is expressed in various tissues in adults, including bone, kidney, and epithelial linings [289,290]. OPN aids in cell migration, adhesion, and survival of various cell types including ECs, SMCs, and inflammatory cells through interactions with integrins (α4β1, α5β1, α8β1, α9β1, αvβ1, αvβ3, αvβ5, and αvβ6) and CD44 [291,292,293,294,295]. OPN expression is normally low but is remarkably upregulated at sites of inflammation and tissue remodeling. OPN is mainly produced by injured and inflamed epithelial cells, ECs, SMCs, and certain tumor cells, as well as by inflammatory infiltrating cells comprising T cells and macrophages [296,297,298,299]. Ang II has been shown to induce OPN expression in cardiac MVECs in a time- and concentration-dependent manner, and this effect was inhibited by treatment with Ang II receptor type 1 antagonist, losartan. Furthermore, inhibition of NOXs with diphenylene iodonium prevented Ang II-induced upregulation of OPN, highlighting the involvement of redox-sensitive signaling in this process [300].

Golledge et al. reported upregulated OPN expression in the proximal segments of atherosclerotic internal carotid arteries isolated from symptomatic patients compared with asymptomatic individuals [301]. Increased OPN levels in symptomatic patients may promote infiltration of macrophages and secretion of proteolytic enzymes, thereby reducing plaque stability [302]. Matsui et al. demonstrated that the deficiency of OPN reduces the size of atherosclerotic lesions in female *Apoe*/OPN double-knockout mice after 36 weeks of a normal chow diet [224]. These findings align with previous findings indicating that OPN attracts macrophages and lymphocytes, therefore, promotes vascular inflammation and atherogenesis [303]. However, no differences in atherosclerosis were observed among male control and OPN-deficient mice. On the other hand, endothelial dysfunction is known to play an important role in the early stages of atherosclerosis. OPN has been associated with reduced eNOS activity within atherosclerotic lesions, exacerbating endothelial dysfunction in individuals with CAD [304,305]. OPN overexpression in mice stimulates medial thickening, neointima formation, and accelerates atherosclerosis progression [224,306]. Moreover, transgenic mice overexpressing OPN exhibit accelerated development of fatty streaks and mononuclear cell-rich lesions, resulting in larger atherosclerotic plaques compared with control mice after being fed an atherogenic diet [306,307], suggesting the pro-atherogenic role of OPN.

OPN, via binding to α9β1 integrin, has been shown to activate ERK and p38 signaling pathways, which stimulate COX-2 expression in macrophages and promote angiogenesis [308]. OPN plays a significant role in orchestrating the inflammatory response and modulating EC function. Dai et al. reported that OPN promotes EC proliferation, migration, and tube formation, and induces activation of PI3K/AKT- and ERK-mediated pathways [309]. Further, OPN treatment stimulated VEGF mRNA and protein expression in dose- and time-dependent manners responsible for the second phase of AKT and ERK signaling. Additional in vivo experiments showed inhibition of angiogenesis with anti-OPN antibody treatment in the corneal pocket and MCF-7 xenograft models [309]. Furthermore, in vitro studies have highlighted the significant role of OPN in the chemotaxis of inflammatory cells [296,310]. Additionally, OPN stimulates EC proliferation via inducing tumor cells to release VEGF. OPN present in tumors interacts with CD44 and integrin receptors, thereby facilitating PI3K/Akt/eNOS- signaling, which in turn promotes EC proliferation, migration, and tube formation in vitro [311,312,313]. These cellular changes promote angiogenesis within tumors and stimulate tumor growth. Lyle et al. revealed that H_2_O_2_ stimulates OPN translation via phosphorylating 4E-BP1 (Ser65) in VSMCs [314]. Further in vivo study by the same group demonstrated increased OPN and H_2_O_2_ levels in ligated femoral arteries compared with controls [315] and scavenging of H_2_O_2_ with polyethylene glycol-catalase infusion and SMC-specific catalase overexpression blunted ischemia-induced OPN, suggesting a crucial role of H_2_O_2_ in the induction of OPN expression during ischemia. Additionally, the reduction in OPN expression resulted in impaired neovascularization [315]. Altogether, OPN plays a multifaceted role in EC inflammation and atherosclerosis, and further research is required to fully understand the underlying molecular mechanisms.

### 4.3. Roof Plate-Specific Spondins (RSPOs)

Roof plate-specific spondins (RSPOs) are involved in many biological processes, such as tissue homeostasis, stem cell control, and embryonic development [11,316,317,318,319]. There are four types of RSPOs, namely RSPO1, RSPO2, RSPO3, and RSPO4, all of which are crucial in regulating Wnt/β-catenin signaling, essential for various developmental processes and tissue regeneration [11]. RSPO3 has been shown to regulate angiogenesis and vascular remodeling [222]. Different RSPOs function by interacting with various receptors and co-receptors, including leucine-rich repeat-containing G-protein coupled receptors (LGR4, 5 and 6), ZNRF3, and RNF43, and modulate Wnt/β-catenin signaling [318,320,321,322]. This Wnt signaling, in turn, regulates key cellular functions such as proliferation and differentiation, thereby influencing vascular development and angiogenesis [323]. Bretón-Romero et al. have shown that ECs isolated from diabetic patients exhibit higher levels of Wnt5a, have increased JNK activation, and impaired eNOS phosphorylation [324]. Inhibition of Wnt5a and JNK restores eNOS activation and EC function, suggesting the detrimental role of Wnt5a/JNK pathway in endothelial dysfunction during diabetic conditions [324]. Stimulation of human aortic ECs with Wnt5a induces COX-2 expression and elevates inflammatory cytokine levels, while Wnt3a has limited effects, pointing to the role of β-catenin-independent Wnt signaling in inflammatory endothelial activation [325]. Further experiments revealed that Wnt5a increases endothelial permeability and promotes EC invasion, reflecting an inflammatory state of the endothelium. Collectively, this information suggests that Wnt5a plays a significant role in regulating endothelial permeability [324,325,326,327,328].

RSPO1 is one of the extensively studied members of the RSPO family. It aids in embryonic development, particularly in limb formation, hair follicle development, and sex determination. In the zebrafish model, RSPO1-Wnt signaling promotes angiogenesis by stimulating the Vegfc-Vegfr3 axis during development [329]. In line with this, blocking Rspo1 function through genetic mutation or morpholino-mediated *Rspo1* knockdown suppresses angiogenic proliferation and leads to defective trunk and cranial vessel angiogenesis. Moreover, deficiency of the putative Rspo1 receptor Kremen, which is expressed in the vasculature, also results in angiogenic defects in zebrafish [329,330]. Furthermore, Caruso et al. reported that administration of R-spondin 1 rescues Dickkopf-1-induced inhibition of testicular angiogenesis during embryonic development [331]. Additionally, Rspo1/Wnt/β-catenin signaling has been demonstrated to upregulate VEGFaa expression and promote N-methyl-N-nitrosourea-induced abnormal angiogenesis in zebrafish [221]. However, the specific role of RSPO1 in endothelial inflammation remains understudied, given the pleiotropic role of Wnt/β-catenin signaling, RSPO1 may regulate EC inflammation and modulate atherosclerosis development.

**Figure 3:**
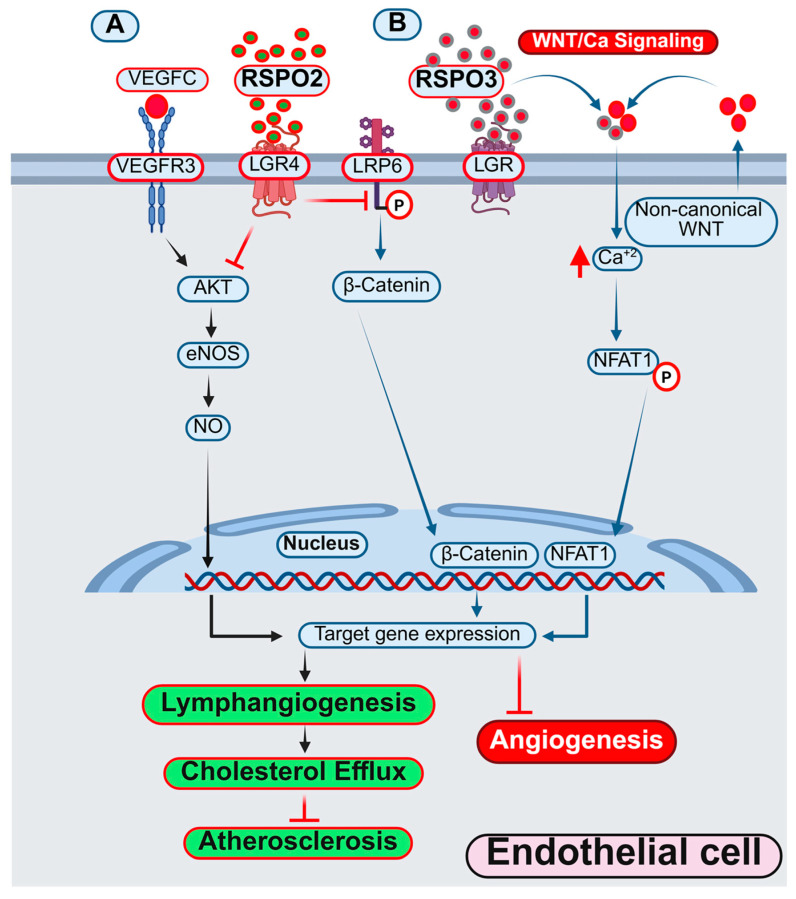
Role of RSPOs in endothelial dysfunction: Schematic representation of various receptors present on ECs that interact with RSPO2 and RSPO3, and their signaling pathways. (**A**) RSPO2 binds to LGR4 receptors on LECs. This interaction inhibits VEGF-C-induced activation of AKT and eNOS, reducing NO production and impairing lymphatic vessel formation. Furthermore, RSPO2 inhibits the canonical Wnt-β-catenin pathway in LECs in a NO-dependent manner, decreasing lymphatic vessel-mediated LDL drainage from arterial walls and contributing to atherosclerosis development. (**B**) RSPO3 activates non-canonical Wnt signaling, including WNT/Ca2+/NFAT, which is crucial for vascular remodeling and inhibits angiogenesis. ↑ (red): increased signaling/expression; Ⱶ (red): inhibition.

In a recent study from our laboratory, we investigated the role of RSPO2 in arterial lymphangiogenesis and atherosclerotic lesion formation [220]. We discovered that RSPO2 negatively regulates VEGFC-VEGFR3-induced lymphangiogenesis both in vitro and in vivo. RSPO2 induces its anti-lymphangiogenic effects via activation of LGR4 receptor, which inhibits PI3K-AKT-eNOS signaling and suppresses canonical Wnt-β-catenin cascade [220] (Figure 3A). Interestingly, we observed an inhibitory role of RSPO2 in LECs on β-catenin activation. Furthermore, investigations into the significance of RSPO2-regulated lymphangiogenesis in atherosclerosis revealed that blockade of LGR4-mediated signaling employing the perivascular application of RSPO2’s decoy receptor (LGR4 extracellular domain) promotes arterial lymphatic vessel density, increases arterial cholesterol drainage and reduces atherosclerosis (Figure 3A). These findings suggest that the inhibition of RSPO2-induced signaling may be a therapeutic target to promote lymphangiogenesis and suppress atherosclerosis [220]. In the same study, we reported that RSPO2 inhibits lymphangiogenesis via impaired NO biogenesis rather than increased ROS-mediated impairment of NO bioavailability [220]. While *Rspo2* deletion in widespread cells utilizing the *UBC*-Cre-*ER^T2^* mouse line increased neutrophil migration into the alveolar space and induced lung permeability in *Rspo2* knockout mice compared with control mice [332], hinting at a beneficial role of *Rspo2* in regulating blood vessel permeability or promoting neutrophil migration. Similarly, Carmon et al. observed the activation of Wnt signaling following RSPO2 exposure across various cell types, including MDCK, HEK293T, and HeLa cells. These findings suggest that RSPO2’s effects on the Wnt-β-catenin pathway are cell-specific [333]. In addition, NF-κB activation has been shown to regulate RSPO2 expression in processes like heterotopic ossification [334,335], and NF-κB signaling pathway plays a significant role in EC activation and atherogenesis [336]. However, it remains to be investigated whether RSPO2 mediates NF-κB-induced EC dysfunction and impaired endothelium permeability. Additionally, Aithabathula et al. recently identified RSPO2 as a key suppressor of hepatic steatosis and fibrosis [337], which are closely associated with the development of atherosclerosis. Therefore, it is tempting to speculate on the beneficial role of hepatocyte RSPO2 in atherosclerosis, in contrast to its detrimental role in vascular cells.

Skaria et al. demonstrated that human EC monolayers treated with RSPO3 have increased permeability compared with vehicle-treated cell layers [338]. This increased permeability is attributable to the induction of intercellular gaps, resulting from the disruption of β-catenin and VE-cadherin alignment at adherent junctions [338]. Conversely, a study by Scholz and colleagues demonstrated RSPO3 as an inducer of Wnt signaling in ECs [222]. They observed that inducible deletion of *Rspo3* results in perturbed developmental and tumor vascular remodeling, leading to reduced microvessel density in deficient mice (Figure 3B). Besides, EC-specific *Rspo3*-deficient mice phenocopied vascular defects observed in non-canonical Wnt signaling factor *Evi/Wls* knockout mice [222]. These results demonstrate the crucial function of RSPO3 in preserving vascular integrity and suggest it as a potential therapeutic target for pathologies associated with aberrant angiogenesis and vessel regression. The downstream effects of RSPO-induced signaling, particularly in ECs, primarily occur through the activation of the Wnt/β-catenin signaling pathway. However, while these effects are well-documented, the precise mechanisms by which RSPOs activate the Wnt/β-catenin signaling pathway are still not fully understood.

### 4.4. Tenascins

The tenascin family comprises four members: tenascin-C (TN-C), tenascin-R (TN-R), tenascin-X (TN-X), and tenascin-W (TN-W). They play important roles in tissue formation, homeostasis, wound healing, and various pathological processes [339,340,341]. TN-R is predominantly expressed in the central nervous system (CNS), especially in areas of axonal growth and guidance during development. While its expression is low in adult CNS, it increases in response to injury or inflammation, suggesting a potential role of TN-R in neural repair [342,343,344,345,346]. TN-X, primarily found in connective tissues, maintains the structural integrity of the ECM and regulates collagen fibrillogenesis. Further, TN-X induces inflammation via activation of TGF-β signaling and interaction with cell surface integrins (α11β1), altering tissue dynamics and immune responses [339,347,348]. TN-W is expressed in various tissues, including the nervous system and skeletal muscle. In brain tumors, TN-W is specifically expressed in blood vessels. Moreover, TN-W has pro-angiogenic activity as demonstrated by in vitro studies using EC cultures [349].

TN-C expression is highly expressed in VSMCs during embryonic development and its levels are also elevated in adults during tissue injury, inflammation, or remodeling [350,351,352]. High expression of TN-C has been linked with several arterial pathologies including intimal hyperplasia, pulmonary artery hypertension, aortic aneurysm/dissection, and cerebral vasospasm [353]. TN-C regulates a wide range of cellular processes, including adhesion, migration, proliferation, and differentiation, which are mediated via its interactions with cell surface receptors and ECM components.

Serum levels of TN-C are significantly higher in patients with CAD compared to non-CAD patients, and an association between TN-C levels and the severity of atherosclerosis was observed, indicating its involvement in lesion formation and disease progression [354,355,356]. Elevated plasma TN-C levels are also associated with a higher incidence of cardiovascular events and increased overall and cardiovascular-related mortality in patients with chronic kidney disease, indicating its potential as a prognostic marker in high-risk populations. Exosomes isolated from CAD patients also exhibit higher levels of TN-C compared to those from non-CAD patients, suggesting a potential role of TN-C in disease pathogenesis and intercellular/interorgan communication [357]. In atheroprone *Apoe^−^*^/*−*^ mice, TN-C expression increases with the progression of atherosclerosis, suggesting its involvement in disease development [358,359]. Additionally, TN-C expression is upregulated in neointimal lesions following balloon catheter-induced vascular injury, highlighting its potential involvement in vascular remodeling after injury [360].

Interestingly, Rupp et al. demonstrated higher sprouting of aortic rings from TN-C-deficient mice in comparison to rings from wild-type mice [361]. TN-C exposure represses EC tube formation, migration, and invasion and induces cell apoptosis via inhibition of YAP signaling in vitro. Elevated TN-C levels are also associated with increased tumor cell survival, enhanced angiogenesis, and higher vessel leakiness, thereby promoting metastasis [362]. Furthermore, in vitro studies demonstrate that treatment with an inflammatory agent LPS, augments TN-C expression in THP-1 macrophages in a dose- and time-dependent manner, and TN-C mediates LPS-induced foam cell formation [363]. Consistently, TN-C is involved in oxLDL-stimulated foam cell formation in THP-1 macrophages, which is abrogated by *CD36* gene silencing and blockade of TLR4, suggesting its role in macrophage lipid uptake and atherogenesis [364]. Similarly, perivascular macrophages activated by TN-C via TLR4 activation release NO and TNF, inducing niche component expression in ECs [365].

**Figure 4:**
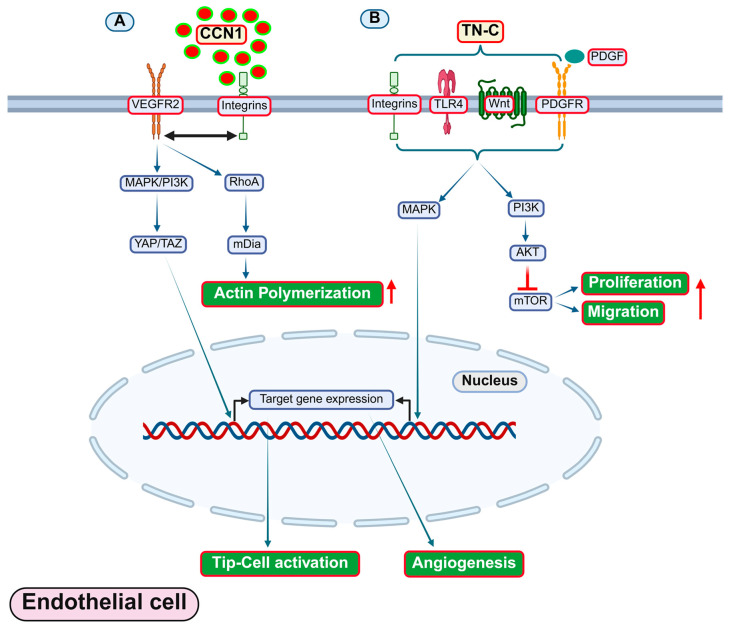
Role of CCN1- and TN-C-regulated signaling in angiogenesis: (**A**) The interaction between CCN1 and αvβ3 activates VEGFR2, leading to downstream MAPK/PI3K-YAP/TAZ signaling and regulation of actin polymerization via Rho effector mDia1. (**B**) TN-C interaction with various cell surface receptors and ECM components. Signaling via integrins, TLR4, and Wnt receptors to intracellular effectors such as MAPK leads to changes in gene transcription, resulting in altered expression of proteins involved in proliferation, adhesion, and cell survival/apoptotic responses. ↑ (red): increased signaling/expression; ↔ (black): interaction; Ⱶ (red): inhibition.

Fibronectin and TN-C, important components of the cell matrix, exhibit pro- and anti-adhesive properties, respectively. TN-C induces fibronectin expression in ECs, which counteracts the anti-adhesive effects of TN-C responsible for reduced EC motility. Consequently, this triggers the assembly of a dense, highly branched subendothelial matrix that promotes tubulogenic activity [366]. Contrary to this, increased lymphangiogenesis was observed following the downregulation of TNC expression. TN-C demonstrated a modest downregulation of genes associated with nuclear division, cell division, and cell migration in lymphatic ECs, suggesting its inhibitory effects on these cellular processes [367] (Figure 4B). Additionally, TN-C is an important inducer of neuroinflammatory cascades and the resultant pathology of stroke. It is upregulated in cerebral arteries and brain tissues including astrocytes, neurons, and brain capillary ECs following subarachnoid hemorrhage [368]. Overall, the above studies suggest that TN-C plays a significant role in regulating angiogenesis, EC function and development. However, the exact mechanisms of how TN-C interacts with ECs and its overall impact on endothelial function require further investigation. 

### 4.5. CCN Proteins

CCN family members are involved in the regulation of cell adhesion, proliferation, migration, differentiation, survival, angiogenesis, and wound healing. This family includes six members named CCN1 to CCN6. CCN proteins play a crucial role as adaptor molecules in multiple cellular processes involved in vascular development and vascular diseases such as atherosclerosis and restenosis. One of their important functions is to bind growth factors like VEGF and TGF-β and facilitate their localization near the cell surface through interactions with cell surface binding partners like integrins or heparan sulfate proteoglycans. CCN2 interacts with various cell surface receptors, including heparan sulfate proteoglycans, tropomyosin-related kinase A (TrkA), and LRP-1 [369,370,371]. On the other hand, CCN3 exerts its effects through interactions with notch receptors and integrins, specifically α6β1 and α6β5 [372,373,374]. CCN5 is mainly expressed in ECs, fibroblasts, and VSMCs within differentiating scar tissue and known to inhibit vascular cell growth and migration, indicating a potential anti-inflammatory protective role [375,376,377,378]. The expression of CCN proteins in vascular endothelium is regulated by shear stress, a mechanical force generated by blood flow in blood vessels [371,379,380,381,382,383,384].

As we know, atherosclerosis is a focal arterial disease, mainly manifesting at the sites of disturbed blood flow, including the lesser curvature of the arch and bifurcations. Human ECs, when exposed to OSS, mimicking disturbed flow at atherosclerotic sites, have increased CCN1 expression [371]. Elevated CCN1 levels contribute to endothelial dysfunction and promote atherosclerosis. The interaction between CCN1 and integrin α6β1 activates NF-κB, leading to increased CCN1 production, thus establishing a positive feedback loop that exacerbates endothelial activation and atherogenesis [385]. Hsu et al. reported that disrupting CCN1-integrin α6β1 interaction by a mutation (defective in binding α6β1) in *Ccn1* gene or using an antagonist T1 peptide (derived from an α6β1-binding sequence of CCN1) may serve as a promising therapeutic strategy to prevent or treat atherosclerosis [385]. Knock-in mice (*Ccn1^dm^*^/*dm*^*/Apoe^−^*^/*−*^) carrying a mutant *Ccn1* allele unable to bind integrin α6β1 exhibit reduced plaque formation, suggesting the importance of CCN1 and integrin α6β1 interaction in atherosclerosis development [385]. Moreover, experimental strategies to manipulate CCN1-induced signaling using gain-/loss-of-function assays in cultured macrophages have revealed that CCN1 promotes lipid accumulation and foam cell formation [226]. In *Apoe^−^*^/*−*^ mice, CCN1 treatment exacerbates atherosclerosis and induces systemic inflammation [226]. Mechanistically, CCN1 treatment downregulates hepatic expression of key genes involved in cholesterol metabolism, including ABCG5, ABCG8, liver X receptor α, cholesterol 7a-hydrolase, and LDL receptor [226]. These findings indicate that CCN1 via increasing hepatic lipid accumulation and inhibiting macrophage cholesterol efflux, promotes atherosclerotic lesion formation. Similar to reduced atherosclerosis in *Ccn1^dm^*^/*dm*^ mice, *Ccn1^dm^*^/*dm*^ mice are resistant to isoproterenol-induced cardiac injury and autophagy. CCN1/α6β1 interaction in cardiomyocytes promotes autophagy via ROS production and activation of ERK and JNK [386], further implicating CCN1 in the pathogenesis of CVDs and highlighting its potential as a target for therapeutic intervention.

CCN proteins stimulate angiogenesis, not only by promoting the migration, adhesion, and survival of vascular ECs but also by facilitating cell communication networks, which centralize the coordination of various growth factors and proteins, and ultimately induce the formation of new blood vessels [387]. In addition, CCN1 via αvβ3 activates VEGFR2 and downstream MAPK/PI3K signaling pathways, YAP/TAZ, as well as Rho effector mDia1 to enhance tip cell activity and autoregulate its own expression [388] (Figure 4A). Inhibition of αvβ3-mediated signaling represses tip cell number and sprouting in retinas of EC-specific *Ccn1* transgenic mice, and allograft tumors in *Ccn1*-overexpressing mice have hyperactive vascular sprouting [388].

CCN1 activates various signaling pathways, such as ILK/Akt, MEK/ERK, and Wnt/β-catenin axis in various cell types [389,390,391,392]. In ECs, CCN1-induced by OSS promotes superoxide production, NF-κB activation, and expression of inflammatory genes [371,385]. This suggests a pro-inflammatory role of CCN1 in ECs under disturbed flow conditions. In addition, a previous study has demonstrated that the knockdown of *CCN1* in human retinal vascular ECs reduces NOX4 expression and inhibits ROS production [393]. Consistently, treatment with recombinant CCN1 stimulates generation of ROS in human/mouse neutrophils, further supporting its role in promoting oxidative stress [394]. In macrophages, CCN1 interacts with integrin αMβ2 and syndecan-4 to enhance cell adhesion and activate NF-κB-mediated pro-inflammatory responses as evidenced by elevated levels of TNF-α, IL-1α, IL-1β, IL-6, and IL-12b [395]. CCN1 is also reported to be expressed in VSMCs of atheromatous plaques, where its expression is regulated by Ang II [379]. Consistently, *CCN1* knockdown significantly suppresses neointimal hyperplasia in rats after 14 and 28 days of vascular injury [396]. CCN1 activation has several downstream effects pivotal for inflammation and tissue repair processes. It modulates immune cells (lymphocytes and monocytes) migration in a biphasic manner. Initially, it enhances actin polymerization and transwell migration, but prolonged exposure to CCN1 inhibits immune cell migration by suppressing the activation status of PI3Kγ, p38, and Akt [397]. Furthermore, CCN1 promotes the expression of various pro-inflammatory cytokines and chemokines in macrophages [395]. Collectively, existing literature suggests that novel approaches targeting CCN1 expression or secretion may hold therapeutic potential for treating atherosclerosis by attenuating endothelial dysfunction, foam cell formation, and hepatic lipid accumulation.

### 4.6. Secreted Protein Acidic and Rich in Cysteine

Secreted Protein Acidic and Rich in Cysteine (SPARC), also known as osteonectin (ON) or BM-40, is a calcium-binding glycoprotein [398,399,400]. It interacts with various components of the ECM, including collagen and fibronectin, and is found in several tissues and organs, such as bone, cartilage, skin, and the ECM of tumors [401,402]. Recent studies have implicated SPARC in vascular pathologies. Hu et al. reported elevated expression of SPARC in VSMCs of atherosclerotic rats and demonstrated a reduction in its expression with aerobic exercise [403]. Similarly, Li et al. observed higher serum levels of SPARC in hypertensive rats, with a positive correlation between SPARC levels and elevated BP [46]. Mechanistically, SPARC has been shown to inhibit endothelium-dependent vessel relaxation, suggesting a potential involvement in vascular dysfunction. However, the precise role of SPARC in atherosclerosis development, including its cell-specific functions, is far from being completely understood.

SPARC is known to modulate various cellular processes in ECs, including adhesion, migration, proliferation, and differentiation, consequently affecting angiogenesis via regulating responses to different growth factors [404,405,406,407,408]. Growth factors like TGF-β, PDGF, and IGF-1 have been demonstrated to stimulate the synthesis of SPARC in chondrocytes [401]. Additional experiments have shown that these growth factors rescue from the inhibitory effects of IL-1 on SPARC synthesis. Similarly, Sage et al. reported increased SPARC levels in aortic ECs exposed to endotoxin and an association between SPARC levels and proliferative cells in vivo, suggesting its cell proliferative effects [408]. Conversely, SPARC has been found to inhibit DNA synthesis and suppress ERK1/2 activation in VEGF-treated ECs by directly binding to VEGF and preventing its interaction with its cell surface receptors [407]. Other studies have also revealed similar findings that SPARC suppresses EC cycle progression [409], inhibits binding of PDGF-AB and -BB to its receptors [410], and regulate EC morphology and barrier function [411]. Interestingly, the role of SPARC has also been investigated under oxidative stress conditions within the tumor microenvironment. Studies using HUVECs have shown that SPARC expression is downregulated in the presence of H_2_O_2_ compared to control conditions; however, SPARC levels can be restored upon ROS inhibition with diphenyleneiodonium [412].

SPARC expression in cultured human cerebral MVECs positively correlates with cell proliferation and reduces as these cells mature and form a confluent monolayer [413]. Besides, treatment with recombinant SPARC increases permeability and decreases transendothelial electrical resistance. These findings indicate that SPARC may significantly affect the function of cerebral microvessels during development and inflammation at the blood–brain barrier. In another study, it was reported that the deficiency of SPARC inhibits endoglin-mediated pericyte migration, alters endoglin binding in focal complexes, increases TGF-β activation via αV integrin pathway, and results in decreased pericyte-associated vessels in an orthotopic model of pancreatic cancer [414]. These results highlight the function of SPARC in promoting pericyte migration through TGF-β. Further, these studies provide valuable insights into the role of SPARC in EC inflammation and its potential effects on vascular diseases and repair mechanisms. As described above, SPARC exhibits an anti-angiogenic role by either impeding the interaction of growth factors with their cell surface receptors or modulating the expression of MMPs and TGF-β1 [415,416,417]. This anti-angiogenic function is abolished in SPARC knockout mice [404,407]. Conversely, certain cleavage products of SPARC or intact SPARC have been observed to promote angiogenesis [408,418].

## 5. Therapeutic Potential of Targeting Different Matricellular Proteins

Matricellular proteins are minimally expressed in healthy adult tissues but get upregulated in response to stress, injury, or disease. As described above, various matricellular proteins have been demonstrated to promote atherosclerosis via stimulating vascular inflammation and regulating the phenotype of different vascular and immune cells involved in its pathogenesis. Therefore, targeting the signaling mechanisms induced by these proteins and/or regulating their expression may offer significant therapeutic potential to reduce and even regress plaque progression.

TSP1 is a well-known antiangiogenic matricellular protein that can either promote or inhibit inflammation, depending on the cellular context. In atherosclerosis, its therapeutic relevance primarily lies in the inhibition of TSP1-induced CD47 activation. TSP1 via CD47 activation in LECs inhibits lymphangiogenesis [246]. Earlier studies have demonstrated that lymphatic vasculature present in the adventitial layer of the arterial wall represents the primary route of cholesterol removal from atherosclerotic vessels, and improved lymphatic function leads to atherosclerosis regression [419,420]. We have previously demonstrated that blockade of TSP1-induced CD47 activation in LECs promotes in vitro lymphangiogenesis and mice with LEC-restricted *Cd47* deletion have enhanced arterial lymphatic vessel density and attenuated atherosclerosis [246]. This signaling axis also promotes EC senescence, contributing to vascular aging and dysfunction [245], thereby, inhibiting TSP1-CD47 interactions may help reverse age-related endothelial impairments and atherosclerosis. Additionally, TSP1 suppresses NO production, a key regulator of vasodilation and vascular homeostasis. Therapeutic strategies targeting TSP1 to restore NO bioavailability represent another promising approach to combat vascular inflammation and dysfunction [273]. This information supports the therapeutic potential of blocking the TSP1-CD47 axis for preventing vascular inflammation, compromised endothelial barrier integrity, and atherosclerosis. Consistently, Kojima et al. demonstrated that a CD47-blocking antibody originally developed as an anti-cancer drug improves efferocytic removal of apoptotic cells from the vascular wall, leading to attenuated vascular inflammation and atherosclerosis in mice [421]. Jarr et al. reported in a phase 1b-2 trial that a humanized anti-CD47 antibody magrolimab induces tumor reduction in patients with relapsed or refractory lymphoma or acute myeloid leukemia (AML) [422]. Additionally, the authors reported reduced vascular inflammation in those patients as determined by ^18^F-fluorodeoxyglucose uptake in carotid arteries. Several other CD47-blocking antibodies are currently in various stages of clinical trials targeting different types of cancer (Supplementary Table S1) [423,424]. In addition, macrophage-specific nanotherapy approaches have been explored to inhibit the CD47-SIRPα antiphagocytic signaling axis [425]. For example, a novel nanotherapy using single-walled carbon nanotubes (SWNTs) loaded with an SHP1 inhibitor (SWNT-SHP1i) has been developed for systemic blockade of CD47 signaling. This approach was tested in a porcine model (large animal model) of early atherosclerosis [426], demonstrating the potential for targeted therapeutic strategies aimed at enhancing efferocytosis in atherosclerotic cardiovascular disease.

Singla et al. demonstrated that blockade of RSPO2–LGR4 signaling via perivascular application of Rspo2’s decoy receptor attenuates atherosclerosis. A mouse LGR4/Gpr48 Fc chimeric protein, containing the N-terminal extracellular domain (ECD) of LGR4, was used for this purpose. The ECD of LGR4 is the recognized binding site for RSPO ligands, and LGR4-ECD has been shown to effectively inhibit LGR4 signaling both in vitro and in vivo [220]. The antibodies 130M23, 130M24, 130M25, 130M26, 130M27, and 130M28 are specific inhibitors of RSPO2 and have been shown to reduce or completely block RSPO2-induced β-catenin signaling (Patent No.: US 9,644,034). These antibodies may serve as blocking agents to disrupt RSPO2-LGR4 interactions. Additionally, inhibition of Wnt signaling using small molecule inhibitors, recombinant proteins, or neutralizing antibodies has been shown to ameliorate atherosclerosis in preclinical models. For example, the porcupine inhibitor GNF-6231 and XAV939, small molecules that inhibit poly ADP-ribose polymerase tankyrase 1 and tankyrase 2, have demonstrated efficacy in this context. Similarly, LGK974 and SFRP4 are reported to block Wnt/β-catenin signaling [427]. Therefore, inhibition of Rspo2-induced LGR4 activation and downstream Wnt signaling utilizing recombinant proteins, blocking antibodies, and small molecule inhibitors has the potential to reduce atherosclerosis; however, this possibility requires further investigation.

OPN contains an RGD sequence that binds to integrins and CD44 on monocytes, macrophages, and immune cells, promoting their recruitment to plaques. This interaction drives macrophage activation, foam cell formation, and VSMCs migration, contributing to inflammation and plaque progression [428]. Blocking OPN-integrin binding reduces its pro-inflammatory effects. While primarily pro-atherogenic, OPN also inhibits vascular calcification, which can paradoxically stabilize plaques. Thus, OPN-targeted therapies should be able to balance suppress the inflammation while preserving OPN’s anti-calcific role [429]. Targeted drug delivery using nanoparticles, for example, those carrying the PPARδ agonist GW1516, has shown promise in reducing VSMC migration and apoptosis [430].

TN-C levels are upregulated in atherosclerotic lesions. Several peptides and vector fragments, including G11, G11-iRGD, TN11, PL1, and PL3 have been developed that recognize TN-C domains and can be used to direct therapeutic or diagnostic payloads to plaque tissue. Furthermore, ATN-RNA and IMA950 were investigated in clinical trials as therapeutic drugs and vaccines by targeting TN-C, respectively [431]. In other cardiovascular-remodeling settings (post-myocardial infarction), modulating TN-C may also be relevant—either inhibiting its excessive up-regulation (which promotes adverse ventricular remodeling via macrophage polarization to a pro-inflammatory M1 phenotype through TLR4) or harnessing its transient, wound-healing role during acute repair.

In short, a defining feature of these matricellular proteins is their context-dependent dual role in tissue remodeling, inflammation, and repair. Their biological (protective or pathogenic) effects are highly regulated by the cellular microenvironment, stage of disease, and type/duration of injury. For example, certain matricellular proteins promote cell adhesion, migration, proliferation, and repair during acute tissue injury, thereby supporting wound healing. However, the same molecules may drive fibrosis, chronic inflammation, or atherogenesis when their levels are persistently elevated. For example, TSP1 inhibits angiogenesis by activating TGF-β and suppressing EC proliferation, yet in other settings, it promotes tumor progression by enhancing matrix remodeling, cell migration, and cell invasive behavior. Similarly, OPN contributes to wound healing by promoting cell survival and migration, but its long-term higher expression induces pathological fibrosis, chronic inflammation, and tumor metastasis. These functional differences may arise from the expression levels of various interacting partners (integrins, CD44, CD47, CD36, LRP receptors), post-translational modifications, and crosstalk with other matricellular proteins and signaling molecules. Therefore, a precise temporal and spatial understanding of their protein expression and downstream signaling pathways is required for elucidating their multifaceted roles. Such insights will be critical to design specific targeted therapies to promote their protective functions while mitigating their deleterious effects in individual pathologies.

## 6. Conclusions and Future Perspectives

Atherosclerosis is the underlying cause of CVD-related mortality worldwide. Among the various cell types involved in the pathogenesis of atherosclerosis, EC dysfunction triggered by disturbed blood flow or other proatherogenic risk factors is the initiating event in the pathogenesis of atherosclerosis. Matricellular proteins are recognized to play important roles in EC inflammation and the progression of atherosclerosis. Elevated levels of these proteins in arteries correlate with atherosclerosis; however, their precise role, either deleterious or protective during atherogenesis, requires further investigations. In vascular ECs, these proteins promote inflammation by facilitating leukocyte adhesion and transmigration across the endothelium. Moreover, they contribute to the chronic inflammatory environment characteristic of atherosclerosis by inducing pro-inflammatory signaling in ECs and remodeling of ECM. Additionally, these proteins regulate plaque stability by affecting VSMC proliferation and migration, as well as collagen deposition in the fibrous cap. Given the complex pathogenesis of atherosclerosis, further research on how these proteins stimulate EC damage, activation, inflammation, and plaque formation, and identification of involved cell surface receptor(s) is warranted. Novel animal models, including EC-restricted knockout/overexpression mice with/without lineage-tracing capabilities, advanced imaging techniques, and transcriptomic sequencing, would be useful in uncovering the precise mechanisms stimulated by these proteins in ECs during disease progression. Additionally, the development of novel pharmacological small-molecule inhibitors or blocking antibodies is crucial to explore the translational potential of inhibiting identified pathways. While gene-deficient mouse models provide valuable mechanistic insights, they may not fully replicate the outcome of pharmacological inhibition, as small molecules and antibodies may have off-target and adverse effects. Currently, limited information is available on the roles of matricellular proteins in regulating ROS generation, redox-sensitive signaling, and autophagy during endothelial dysfunction; therefore, future studies are warranted on these aspects. Similarly, the effects of matricellular proteins on LDL uptake and clearance by ECs remain underexplored, despite their relevance in atherogenesis.

Matricellular proteins may also play an important role in regulating the liver-vascular axis and consequently governing atherogenesis. Supporting this concept, our lab has recently demonstrated that nidogen-2 (NID2) levels are upregulated in both human atherosclerotic arteries and murine steatotic livers [432]. Adeno-associated virus (AAV)-mediated overexpression of *NID2* in a mouse model promotes both hepatic steatosis and atherosclerotic lesion formation. Notably, since intraperitoneal injection of AAV primarily induces gene expression in the liver, it is plausible that liver-derived NID2 contributes to the observed vascular phenotype. However, future studies with hepatocyte-specific NID2 deletion/overexpression are required to establish its role in liver–vascular axis. Further, Hepatocyte RSPO2 has been identified as a key suppressor of hepatosteatosis [337], a condition closely associated with atherosclerosis. Therefore, it is tempting to speculate on the beneficial role of hepatocyte RSPO2 in atherosclerosis in contrast to its detrimental role in vascular cells. However, the role of matricellular proteins secreted from the liver and binding to their cognate receptors present on vascular cells remains to be investigated.

To better understand the expression of matricellular proteins in ECs at the single-cell level and across different stages of atherogenesis (early and advanced), studies using global or cell-specific knockout mice, combined with transcriptomic profiling of ECs during inflammation and atherogenesis with single-cell and/or bulk RNA sequencing (RNA-seq) of aortic ECs, are warranted. Some limited RNA-seq studies have been reported, such as those investigating SMCs and atherosclerosis using a CD47 SMC-specific knockout mouse model [433]. However, RNA-seq studies focusing on matricellular proteins, EC inflammation, and atherogenesis remain underexplored.

Despite knowing that matricellular proteins contribute to atherogenesis, targeting these presents significant challenges. They often exhibit both pro- and anti-inflammatory effects depending on their expression level, tissue type, disease stage, and microenvironment. Their expression is transient, spatially restricted, and induced by stress or injury, complicating timing and delivery of therapy. These proteins bind multiple, often weakly interacting partners/receptors (e.g., integrins, CD44, CD47, CD36, growth factors), making it difficult to block specific interactions without off-target effects. Being embedded in the ECM also limits drug accessibility, and large molecules, such as blocking antibodies, may not penetrate enough dense stroma to inhibit the respective signaling. Finally, as key mediators of tissue repair and remodeling, their inhibition risks disrupting normal physiological processes. These drug development challenges may be overcome with different potential strategies: (i) Antibody-drug conjugates or targeted delivery systems to selectively deliver therapeutics to matricellular protein-expressing tissues. (ii) Peptide inhibitors or mimetics that block specific binding domains. (iii) RNA-based therapeutics (e.g., siRNA, antisense oligonucleotides-ASOs) with potential tissue specificity.

Addressing these knowledge gaps will be useful in advancing our understanding of matricellular protein biology and for developing effective therapeutic strategies for CVDs.

## Data Availability

No new data were created or analyzed in this study. Data sharing does not apply to this article.

## Supplementary Materials

The following supporting information can be downloaded at: https://www.mdpi.com/article/10.3390/antiox14111338/s1, Table S1. Overview of clinical trials on various CD47-blocking antibodies.

## Abbreviations

ECM	Extracellular Matrix
EC/ECs	Endothelial Cells
CVDs	Cardiovascular Diseases
LDL	Low-Density Lipoprotein
VSMCs	Vascular Smooth Muscle Cells
CAD	Coronary Artery Disease
NO	Nitric Oxide
TNF-α	Tumor Necrosis Factor-alpha
IL-1	Interleukin-1
ICAM-1	Intercellular Adhesion Molecule-1
VCAM-1	Vascular Cell Adhesion Molecule-1
NF-κB	Nuclear Factor-Kappa B
MAPK	Mitogen-Activated Protein Kinase
MCP-1	Monocyte Chemoattractant Protein-1
EndMT	Endothelial-to-Mesenchymal Transition
ROS	Reactive oxygen species
NOXs	NADPH Oxidases
O2•−	Superoxide Ion
SOD	Superoxide Dismutase
TSPs	Thrombospondins
OPN	Osteopontin
RSPOs	Roof Plate-Specific Spondins
TN-C	Tenascin-C
SPARC	Secreted Protein Acidic and Rich in Cysteine
HUVECs	Human Umbilical Vein ECs
AML	Acute Myeloid Leukemia
