# Supplementary material for: Role of Matricellular Proteins in Endothelial Cell Inflammation and Atherosclerosis
Source: Antioxidants (Basel). 2025 Nov 6;14(11):1338. doi: 10.3390/antiox14111338 (PMC12649302; doi:10.3390/antiox14111338)
Supplement: Supplementary file 1 [file antioxidants-14-01338-s001.zip › antioxidants-3927678-supplementary.pdf]

Supplementary Table 1. Overview of clinical trials on various CD47-blocking antibodies.

Agent	Regimen	Mechanism	Selected population for trials	Clinical trials ID	Company
**AO-176** (Humanized IgG2 anti-CD47 mAb)	AO-176 OR AO-176 + paclitaxel OR AO-176 + pembrolizumab AO-176 OR AO-176 + either dexamethasone OR dexamethasone + bortezomib	Designed for minimal red blood cell binding, preferential tumor cell binding (acidic microenvironment), and some direct tumor-cell kill (not strictly ADCC).	Solid tumors, multiple myeloma (phase I/II)	NCT03834948<br><br>NCT04445701	Arch Oncology
**Magrolimab** (also known as Hu5F9-G4) (Humanized IgG4 anti-CD47 mAb)	Magrolimab + daratumumab + pomalidomide + dexamethasone + bortezomib<br><br>Magrolimab OR magrolimab + azacitidine<br><br>Magrolimab + rituximab OR rituximab + gemcitabine + oxaliplatin<br><br>Magrolimab + pembrolizumab + 5-FU + platinum OR magrolimab + docetaxe<br><br>Magrolimab + docetaxel OR Magrolimab + rituximab<br><br>Magrolimab + azacitidine OR venetoclax + azacididine	Blocks CD47 “don’t eat me” signal on cancer cells, enabling macrophage phagocytosis; built-in “priming” dose strategy because CD47 on RBCs leads to their efferocytic removal.	Hematologic malignancies (AML, MDS), solid tumors, NHL, and HNSCC.<br>(phase I)<br><br>(phase I/II)<br><br>(phase II)<br><br>(phase II)<br><br>(phase III)	NCT04892446<br><br>NCT03248479<br>NCT02953509<br><br>NCT04854499<br><br>NCT04827576<br><br>NCT04778397<br>NCT04313881<br><br>NCT04788043	Gilead Sciences<br><br>Stanford University, Merck Sharp &

 https://doi.org/10.3390/xxxxx

	Magrolimab + pembrolizumab		Hodgkin's lymphoma (phase II)		Dohme Corp.
**AK117** (Also known as Ligufalimab) (Humanized IgG4 anti-CD47 mAb)	AK117 monotherapy<br><br>AK117 + Azacitidine	Demonstrates strong CD47 binding/blockade, with significantly lower hemagglutination and erythro-phagocytosis compared to Magrolimab in non-clinical models.	Solid tumors + hematologic malignancies, (phase I)<br><br>(phase I/II)	NCT04728334<br>NCT04349969<br><br>CTR20211305	Akeso
**CC-90002** (Humanized IgG4 anti-CD47 mAb)	CC-90002 + Rituximab	Epitope and scaffold engineered to reduce RBC binding; however, clinical monotherapy results were suboptimal.	Hematologic malignancies	NCT02367194	Celgene
**IBI188** (Also known as Letaplimab) (Humanized IgG4 anti-CD47 mAb)	IBI188 + Azacitidine, OR<br>IBI188 + decitabine<br><br>IBI188 Monotherapy, OR<br>IBI188 + rituximab<br><br>Monotherapy	Anti-CD47 mAb aimed for an improved binding profile and tolerability.	Trials in NHL, AML, MDS, advanced solid cancer (phase I/II)<br><br>(phase I)<br><br>(phase I)	CTR20200938<br><br>NCT03717103<br><br>NCT03763149	Innovent Biologics
**DSP-107** (SIRPα - 4-1BBL) (Humanized bi-functional, trimeric, fusion protein-bsAb)	Monotherapy, OR<br>DSP-107 + atezolizumab	A first-in-class CD47 and 4-1BB targeting multifunctional immune-recruitment protein	Advanced solid cancer, non-small cell lung cancer (phase I)	NCT04440735	Kahr medical
**IBI322** (Recombinant anti-human	IBI322 monotherapy, OR	Combining innate-immunity checkpoint (CD47) with adaptive checkpoint (PD-L1) in a single	Advanced malignant tumors,	NCT04328831<br>NCT04912466	Innovent Biologics

CD47/PD-L1 bispecific antibody-bsAb)	IBI322+ azacitidine/decitabine	molecule, promising early responses (~20 % PR in one cohort).	hematologic malignancies that failed standard treatment, locally advanced, unresectable, or metastatic tumors (Phase 1)	NCT04795128<br>NCT05148442	(Suzhou) Co. Ltd.

* AML: Acute myeloid leukemia; MDS: Myelodysplastic syndromes; HNSCC: head and neck squamous cell carcinoma; NHL: Non-Hodgkin lymphoma
